# Search for flavour-changing neutral current top-quark decays to $\varvec{qZ}$ in $\varvec{pp}$ collision data collected with the ATLAS detector at $\varvec{\sqrt{s}=8}$ TeV

**DOI:** 10.1140/epjc/s10052-015-3851-5

**Published:** 2016-01-08

**Authors:** G. Aad, B. Abbott, J. Abdallah, O. Abdinov, R. Aben, M. Abolins, O. S. AbouZeid, H. Abramowicz, H. Abreu, R. Abreu, Y. Abulaiti, B. S. Acharya, L. Adamczyk, D. L. Adams, J. Adelman, S. Adomeit, T. Adye, A. A. Affolder, T. Agatonovic-Jovin, J. Agricola, J. A. Aguilar-Saavedra, S. P. Ahlen, F. Ahmadov, G. Aielli, H. Akerstedt, T. P. A. Åkesson, A. V. Akimov, G. L. Alberghi, J. Albert, S. Albrand, M. J. Alconada Verzini, M. Aleksa, I. N. Aleksandrov, C. Alexa, G. Alexander, T. Alexopoulos, M. Alhroob, G. Alimonti, L. Alio, J. Alison, S. P. Alkire, B. M. M. Allbrooke, P. P. Allport, A. Aloisio, A. Alonso, F. Alonso, C. Alpigiani, A. Altheimer, B. Alvarez Gonzalez, D. Álvarez Piqueras, M. G. Alviggi, B. T. Amadio, K. Amako, Y. Amaral Coutinho, C. Amelung, D. Amidei, S. P. Amor Dos Santos, A. Amorim, S. Amoroso, N. Amram, G. Amundsen, C. Anastopoulos, L. S. Ancu, N. Andari, T. Andeen, C. F. Anders, G. Anders, J. K. Anders, K. J. Anderson, A. Andreazza, V. Andrei, S. Angelidakis, I. Angelozzi, P. Anger, A. Angerami, F. Anghinolfi, A. V. Anisenkov, N. Anjos, A. Annovi, M. Antonelli, A. Antonov, J. Antos, F. Anulli, M. Aoki, L. Aperio Bella, G. Arabidze, Y. Arai, J. P. Araque, A. T. H. Arce, F. A. Arduh, J-F. Arguin, S. Argyropoulos, M. Arik, A. J. Armbruster, O. Arnaez, V. Arnal, H. Arnold, M. Arratia, O. Arslan, A. Artamonov, G. Artoni, S. Asai, N. Asbah, A. Ashkenazi, B. Åsman, L. Asquith, K. Assamagan, R. Astalos, M. Atkinson, N. B. Atlay, K. Augsten, M. Aurousseau, G. Avolio, B. Axen, M. K. Ayoub, G. Azuelos, M. A. Baak, A. E. Baas, M. J. Baca, C. Bacci, H. Bachacou, K. Bachas, M. Backes, M. Backhaus, P. Bagiacchi, P. Bagnaia, Y. Bai, T. Bain, J. T. Baines, O. K. Baker, E. M. Baldin, P. Balek, T. Balestri, F. Balli, E. Banas, Sw. Banerjee, A. A. E. Bannoura, H. S. Bansil, L. Barak, E. L. Barberio, D. Barberis, M. Barbero, T. Barillari, M. Barisonzi, T. Barklow, N. Barlow, S. L. Barnes, B. M. Barnett, R. M. Barnett, Z. Barnovska, A. Baroncelli, G. Barone, A. J. Barr, F. Barreiro, J. Barreiro Guimarães da Costa, R. Bartoldus, A. E. Barton, P. Bartos, A. Basalaev, A. Bassalat, A. Basye, R. L. Bates, S. J. Batista, J. R. Batley, M. Battaglia, M. Bauce, F. Bauer, H. S. Bawa, J. B. Beacham, M. D. Beattie, T. Beau, P. H. Beauchemin, R. Beccherle, P. Bechtle, H. P. Beck, K. Becker, M. Becker, S. Becker, M. Beckingham, C. Becot, A. J. Beddall, A. Beddall, V. A. Bednyakov, C. P. Bee, L. J. Beemster, T. A. Beermann, M. Begel, J. K. Behr, C. Belanger-Champagne, W. H. Bell, G. Bella, L. Bellagamba, A. Bellerive, M. Bellomo, K. Belotskiy, O. Beltramello, O. Benary, D. Benchekroun, M. Bender, K. Bendtz, N. Benekos, Y. Benhammou, E. Benhar Noccioli, J. A. Benitez Garcia, D. P. Benjamin, J. R. Bensinger, S. Bentvelsen, L. Beresford, M. Beretta, D. Berge, E. Bergeaas Kuutmann, N. Berger, F. Berghaus, J. Beringer, C. Bernard, N. R. Bernard, C. Bernius, F. U. Bernlochner, T. Berry, P. Berta, C. Bertella, G. Bertoli, F. Bertolucci, C. Bertsche, D. Bertsche, M. I. Besana, G. J. Besjes, O. Bessidskaia Bylund, M. Bessner, N. Besson, C. Betancourt, S. Bethke, A. J. Bevan, W. Bhimji, R. M. Bianchi, L. Bianchini, M. Bianco, O. Biebel, D. Biedermann, S. P. Bieniek, M. Biglietti, J. Bilbao De Mendizabal, H. Bilokon, M. Bindi, S. Binet, A. Bingul, C. Bini, S. Biondi, C. W. Black, J. E. Black, K. M. Black, D. Blackburn, R. E. Blair, J.-B. Blanchard, J. E. Blanco, T. Blazek, I. Bloch, C. Blocker, W. Blum, U. Blumenschein, G. J. Bobbink, V. S. Bobrovnikov, S. S. Bocchetta, A. Bocci, C. Bock, M. Boehler, J. A. Bogaerts, D. Bogavac, A. G. Bogdanchikov, C. Bohm, V. Boisvert, T. Bold, V. Boldea, A. S. Boldyrev, M. Bomben, M. Bona, M. Boonekamp, A. Borisov, G. Borissov, S. Borroni, J. Bortfeldt, V. Bortolotto, K. Bos, D. Boscherini, M. Bosman, J. Boudreau, J. Bouffard, E. V. Bouhova-Thacker, D. Boumediene, C. Bourdarios, N. Bousson, A. Boveia, J. Boyd, I. R. Boyko, I. Bozic, J. Bracinik, A. Brandt, G. Brandt, O. Brandt, U. Bratzler, B. Brau, J. E. Brau, H. M. Braun, S. F. Brazzale, W. D. Breaden Madden, K. Brendlinger, A. J. Brennan, L. Brenner, R. Brenner, S. Bressler, K. Bristow, T. M. Bristow, D. Britton, D. Britzger, F. M. Brochu, I. Brock, R. Brock, J. Bronner, G. Brooijmans, T. Brooks, W. K. Brooks, J. Brosamer, E. Brost, J. Brown, P. A. Bruckman de Renstrom, D. Bruncko, R. Bruneliere, A. Bruni, G. Bruni, M. Bruschi, N. Bruscino, L. Bryngemark, T. Buanes, Q. Buat, P. Buchholz, A. G. Buckley, S. I. Buda, I. A. Budagov, F. Buehrer, L. Bugge, M. K. Bugge, O. Bulekov, D. Bullock, H. Burckhart, S. Burdin, C. D. Burgard, B. Burghgrave, S. Burke, I. Burmeister, E. Busato, D. Büscher, V. Büscher, P. Bussey, J. M. Butler, A. I. Butt, C. M. Buttar, J. M. Butterworth, P. Butti, W. Buttinger, A. Buzatu, A. R. Buzykaev, S. Cabrera Urbán, D. Caforio, V. M. Cairo, O. Cakir, N. Calace, P. Calafiura, A. Calandri, G. Calderini, P. Calfayan, L. P. Caloba, D. Calvet, S. Calvet, R. Camacho Toro, S. Camarda, P. Camarri, D. Cameron, R. Caminal Armadans, S. Campana, M. Campanelli, A. Campoverde, V. Canale, A. Canepa, M. Cano Bret, J. Cantero, R. Cantrill, T. Cao, M. D. M. Capeans Garrido, I. Caprini, M. Caprini, M. Capua, R. Caputo, R. Cardarelli, F. Cardillo, T. Carli, G. Carlino, L. Carminati, S. Caron, E. Carquin, G. D. Carrillo-Montoya, J. R. Carter, J. Carvalho, D. Casadei, M. P. Casado, M. Casolino, E. Castaneda-Miranda, A. Castelli, V. Castillo Gimenez, N. F. Castro, P. Catastini, A. Catinaccio, J. R. Catmore, A. Cattai, J. Caudron, V. Cavaliere, D. Cavalli, M. Cavalli-Sforza, V. Cavasinni, F. Ceradini, B. C. Cerio, K. Cerny, A. S. Cerqueira, A. Cerri, L. Cerrito, F. Cerutti, M. Cerv, A. Cervelli, S. A. Cetin, A. Chafaq, D. Chakraborty, I. Chalupkova, P. Chang, J. D. Chapman, D. G. Charlton, C. C. Chau, C. A. Chavez Barajas, S. Cheatham, A. Chegwidden, S. Chekanov, S. V. Chekulaev, G. A. Chelkov, M. A. Chelstowska, C. Chen, H. Chen, K. Chen, L. Chen, S. Chen, X. Chen, Y. Chen, H. C. Cheng, Y. Cheng, A. Cheplakov, E. Cheremushkina, R. Cherkaoui El Moursli, V. Chernyatin, E. Cheu, L. Chevalier, V. Chiarella, G. Chiarelli, G. Chiodini, A. S. Chisholm, R. T. Chislett, A. Chitan, M. V. Chizhov, K. Choi, S. Chouridou, B. K. B. Chow, V. Christodoulou, D. Chromek-Burckhart, J. Chudoba, A. J. Chuinard, J. J. Chwastowski, L. Chytka, G. Ciapetti, A. K. Ciftci, D. Cinca, V. Cindro, I. A. Cioara, A. Ciocio, F. Cirotto, Z. H. Citron, M. Ciubancan, A. Clark, B. L. Clark, P. J. Clark, R. N. Clarke, W. Cleland, C. Clement, Y. Coadou, M. Cobal, A. Coccaro, J. Cochran, L. Coffey, J. G. Cogan, L. Colasurdo, B. Cole, S. Cole, A. P. Colijn, J. Collot, T. Colombo, G. Compostella, P. Conde Muiño, E. Coniavitis, S. H. Connell, I. A. Connelly, V. Consorti, S. Constantinescu, C. Conta, G. Conti, F. Conventi, M. Cooke, B. D. Cooper, A. M. Cooper-Sarkar, T. Cornelissen, M. Corradi, F. Corriveau, A. Corso-Radu, A. Cortes-Gonzalez, G. Cortiana, G. Costa, M. J. Costa, D. Costanzo, D. Côté, G. Cottin, G. Cowan, B. E. Cox, K. Cranmer, G. Cree, S. Crépé-Renaudin, F. Crescioli, W. A. Cribbs, M. Crispin Ortuzar, M. Cristinziani, V. Croft, G. Crosetti, T. Cuhadar Donszelmann, J. Cummings, M. Curatolo, C. Cuthbert, H. Czirr, P. Czodrowski, S. D’Auria, M. D’Onofrio, M. J. Da Cunha Sargedas De Sousa, C. Da Via, W. Dabrowski, A. Dafinca, T. Dai, O. Dale, F. Dallaire, C. Dallapiccola, M. Dam, J. R. Dandoy, N. P. Dang, A. C. Daniells, M. Danninger, M. Dano Hoffmann, V. Dao, G. Darbo, S. Darmora, J. Dassoulas, A. Dattagupta, W. Davey, C. David, T. Davidek, E. Davies, M. Davies, P. Davison, Y. Davygora, E. Dawe, I. Dawson, R. K. Daya-Ishmukhametova, K. De, R. de Asmundis, A. De Benedetti, S. De Castro, S. De Cecco, N. De Groot, P. de Jong, H. De la Torre, F. De Lorenzi, D. De Pedis, A. De Salvo, U. De Sanctis, A. De Santo, J. B. De Vivie De Regie, W. J. Dearnaley, R. Debbe, C. Debenedetti, D. V. Dedovich, I. Deigaard, J. Del Peso, T. Del Prete, D. Delgove, F. Deliot, C. M. Delitzsch, M. Deliyergiyev, A. Dell’Acqua, L. Dell’Asta, M. Dell’Orso, M. Della Pietra, D. della Volpe, M. Delmastro, P. A. Delsart, C. Deluca, D. A. DeMarco, S. Demers, M. Demichev, A. Demilly, S. P. Denisov, D. Derendarz, J. E. Derkaoui, F. Derue, P. Dervan, K. Desch, C. Deterre, P. O. Deviveiros, A. Dewhurst, S. Dhaliwal, A. Di Ciaccio, L. Di Ciaccio, A. Di Domenico, C. Di Donato, A. Di Girolamo, B. Di Girolamo, A. Di Mattia, B. Di Micco, R. Di Nardo, A. Di Simone, R. Di Sipio, D. Di Valentino, C. Diaconu, M. Diamond, F. A. Dias, M. A. Diaz, E. B. Diehl, J. Dietrich, S. Diglio, A. Dimitrievska, J. Dingfelder, P. Dita, S. Dita, F. Dittus, F. Djama, T. Djobava, J. I. Djuvsland, M. A. B. do Vale, D. Dobos, M. Dobre, C. Doglioni, T. Dohmae, J. Dolejsi, Z. Dolezal, B. A. Dolgoshein, M. Donadelli, S. Donati, P. Dondero, J. Donini, J. Dopke, A. Doria, M. T. Dova, A. T. Doyle, E. Drechsler, M. Dris, E. Dubreuil, E. Duchovni, G. Duckeck, O. A. Ducu, D. Duda, A. Dudarev, L. Duflot, L. Duguid, M. Dührssen, M. Dunford, H. Duran Yildiz, M. Düren, A. Durglishvili, D. Duschinger, M. Dyndal, C. Eckardt, K. M. Ecker, R. C. Edgar, W. Edson, N. C. Edwards, W. Ehrenfeld, T. Eifert, G. Eigen, K. Einsweiler, T. Ekelof, M. El Kacimi, M. Ellert, S. Elles, F. Ellinghaus, A. A. Elliot, N. Ellis, J. Elmsheuser, M. Elsing, D. Emeliyanov, Y. Enari, O. C. Endner, M. Endo, J. Erdmann, A. Ereditato, G. Ernis, J. Ernst, M. Ernst, S. Errede, E. Ertel, M. Escalier, H. Esch, C. Escobar, B. Esposito, A. I. Etienvre, E. Etzion, H. Evans, A. Ezhilov, L. Fabbri, G. Facini, R. M. Fakhrutdinov, S. Falciano, R. J. Falla, J. Faltova, Y. Fang, M. Fanti, A. Farbin, A. Farilla, T. Farooque, S. Farrell, S. M. Farrington, P. Farthouat, F. Fassi, P. Fassnacht, D. Fassouliotis, M. Faucci Giannelli, A. Favareto, L. Fayard, P. Federic, O. L. Fedin, W. Fedorko, S. Feigl, L. Feligioni, C. Feng, E. J. Feng, H. Feng, A. B. Fenyuk, L. Feremenga, P. Fernandez Martinez, S. Fernandez Perez, J. Ferrando, A. Ferrari, P. Ferrari, R. Ferrari, D. E. Ferreira de Lima, A. Ferrer, D. Ferrere, C. Ferretti, A. Ferretto Parodi, M. Fiascaris, F. Fiedler, A. Filipčič, M. Filipuzzi, F. Filthaut, M. Fincke-Keeler, K. D. Finelli, M. C. N. Fiolhais, L. Fiorini, A. Firan, A. Fischer, C. Fischer, J. Fischer, W. C. Fisher, E. A. Fitzgerald, N. Flaschel, I. Fleck, P. Fleischmann, S. Fleischmann, G. T. Fletcher, G. Fletcher, R. R. M. Fletcher, T. Flick, A. Floderus, L. R. Flores Castillo, M. J. Flowerdew, A. Formica, A. Forti, D. Fournier, H. Fox, S. Fracchia, P. Francavilla, M. Franchini, D. Francis, L. Franconi, M. Franklin, M. Frate, M. Fraternali, D. Freeborn, S. T. French, F. Friedrich, D. Froidevaux, J. A. Frost, C. Fukunaga, E. Fullana Torregrosa, B. G. Fulsom, T. Fusayasu, J. Fuster, C. Gabaldon, O. Gabizon, A. Gabrielli, A. Gabrielli, G. P. Gach, S. Gadatsch, S. Gadomski, G. Gagliardi, P. Gagnon, C. Galea, B. Galhardo, E. J. Gallas, B. J. Gallop, P. Gallus, G. Galster, K. K. Gan, J. Gao, Y. Gao, Y. S. Gao, F. M. Garay Walls, F. Garberson, C. García, J. E. García Navarro, M. Garcia-Sciveres, R. W. Gardner, N. Garelli, V. Garonne, C. Gatti, A. Gaudiello, G. Gaudio, B. Gaur, L. Gauthier, P. Gauzzi, I. L. Gavrilenko, C. Gay, G. Gaycken, E. N. Gazis, P. Ge, Z. Gecse, C. N. P. Gee, Ch. Geich-Gimbel, M. P. Geisler, C. Gemme, M. H. Genest, S. Gentile, M. George, S. George, D. Gerbaudo, A. Gershon, S. Ghasemi, H. Ghazlane, B. Giacobbe, S. Giagu, V. Giangiobbe, P. Giannetti, B. Gibbard, S. M. Gibson, M. Gilchriese, T. P. S. Gillam, D. Gillberg, G. Gilles, D. M. Gingrich, N. Giokaris, M. P. Giordani, F. M. Giorgi, F. M. Giorgi, P. F. Giraud, P. Giromini, D. Giugni, C. Giuliani, M. Giulini, B. K. Gjelsten, S. Gkaitatzis, I. Gkialas, E. L. Gkougkousis, L. K. Gladilin, C. Glasman, J. Glatzer, P. C. F. Glaysher, A. Glazov, M. Goblirsch-Kolb, J. R. Goddard, J. Godlewski, S. Goldfarb, T. Golling, D. Golubkov, A. Gomes, R. Gonçalo, J. Goncalves Pinto Firmino Da Costa, L. Gonella, S. González de la Hoz, G. Gonzalez Parra, S. Gonzalez-Sevilla, L. Goossens, P. A. Gorbounov, H. A. Gordon, I. Gorelov, B. Gorini, E. Gorini, A. Gorišek, E. Gornicki, A. T. Goshaw, C. Gössling, M. I. Gostkin, D. Goujdami, A. G. Goussiou, N. Govender, E. Gozani, H. M. X. Grabas, L. Graber, I. Grabowska-Bold, P. O. J. Gradin, P. Grafström, K-J. Grahn, J. Gramling, E. Gramstad, S. Grancagnolo, V. Gratchev, H. M. Gray, E. Graziani, Z. D. Greenwood, K. Gregersen, I. M. Gregor, P. Grenier, J. Griffiths, A. A. Grillo, K. Grimm, S. Grinstein, Ph. Gris, J.-F. Grivaz, J. P. Grohs, A. Grohsjean, E. Gross, J. Grosse-Knetter, G. C. Grossi, Z. J. Grout, L. Guan, J. Guenther, F. Guescini, D. Guest, O. Gueta, E. Guido, T. Guillemin, S. Guindon, U. Gul, C. Gumpert, J. Guo, Y. Guo, S. Gupta, G. Gustavino, P. Gutierrez, N. G. Gutierrez Ortiz, C. Gutschow, C. Guyot, C. Gwenlan, C. B. Gwilliam, A. Haas, C. Haber, H. K. Hadavand, N. Haddad, P. Haefner, S. Hageböck, Z. Hajduk, H. Hakobyan, M. Haleem, J. Haley, D. Hall, G. Halladjian, G. D. Hallewell, K. Hamacher, P. Hamal, K. Hamano, A. Hamilton, G. N. Hamity, P. G. Hamnett, L. Han, K. Hanagaki, K. Hanawa, M. Hance, P. Hanke, R. Hanna, J. B. Hansen, J. D. Hansen, M. C. Hansen, P. H. Hansen, K. Hara, A. S. Hard, T. Harenberg, F. Hariri, S. Harkusha, R. D. Harrington, P. F. Harrison, F. Hartjes, M. Hasegawa, Y. Hasegawa, A. Hasib, S. Hassani, S. Haug, R. Hauser, L. Hauswald, M. Havranek, C. M. Hawkes, R. J. Hawkings, A. D. Hawkins, T. Hayashi, D. Hayden, C. P. Hays, J. M. Hays, H. S. Hayward, S. J. Haywood, S. J. Head, T. Heck, V. Hedberg, L. Heelan, S. Heim, T. Heim, B. Heinemann, L. Heinrich, J. Hejbal, L. Helary, S. Hellman, D. Hellmich, C. Helsens, J. Henderson, R. C. W. Henderson, Y. Heng, C. Hengler, S. Henkelmann, A. Henrichs, A. M. Henriques Correia, S. Henrot-Versille, G. H. Herbert, Y. Hernández Jiménez, R. Herrberg-Schubert, G. Herten, R. Hertenberger, L. Hervas, G. G. Hesketh, N. P. Hessey, J. W. Hetherly, R. Hickling, E. Higón-Rodriguez, E. Hill, J. C. Hill, K. H. Hiller, S. J. Hillier, I. Hinchliffe, E. Hines, R. R. Hinman, M. Hirose, D. Hirschbuehl, J. Hobbs, N. Hod, M. C. Hodgkinson, P. Hodgson, A. Hoecker, M. R. Hoeferkamp, F. Hoenig, M. Hohlfeld, D. Hohn, T. R. Holmes, M. Homann, T. M. Hong, L. Hooft van Huysduynen, W. H. Hopkins, Y. Horii, A. J. Horton, J-Y. Hostachy, S. Hou, A. Hoummada, J. Howard, J. Howarth, M. Hrabovsky, I. Hristova, J. Hrivnac, T. Hryn’ova, A. Hrynevich, C. Hsu, P. J. Hsu, S.-C. Hsu, D. Hu, Q. Hu, X. Hu, Y. Huang, Z. Hubacek, F. Hubaut, F. Huegging, T. B. Huffman, E. W. Hughes, G. Hughes, M. Huhtinen, T. A. Hülsing, N. Huseynov, J. Huston, J. Huth, G. Iacobucci, G. Iakovidis, I. Ibragimov, L. Iconomidou-Fayard, E. Ideal, Z. Idrissi, P. Iengo, O. Igonkina, T. Iizawa, Y. Ikegami, K. Ikematsu, M. Ikeno, Y. Ilchenko, D. Iliadis, N. Ilic, T. Ince, G. Introzzi, P. Ioannou, M. Iodice, K. Iordanidou, V. Ippolito, A. Irles Quiles, C. Isaksson, M. Ishino, M. Ishitsuka, R. Ishmukhametov, C. Issever, S. Istin, J. M. Iturbe Ponce, R. Iuppa, J. Ivarsson, W. Iwanski, H. Iwasaki, J. M. Izen, V. Izzo, S. Jabbar, B. Jackson, M. Jackson, P. Jackson, M. R. Jaekel, V. Jain, K. Jakobs, S. Jakobsen, T. Jakoubek, J. Jakubek, D. O. Jamin, D. K. Jana, E. Jansen, R. Jansky, J. Janssen, M. Janus, G. Jarlskog, N. Javadov, T. Javůrek, L. Jeanty, J. Jejelava, G.-Y. Jeng, D. Jennens, P. Jenni, J. Jentzsch, C. Jeske, S. Jézéquel, H. Ji, J. Jia, Y. Jiang, S. Jiggins, J. Jimenez Pena, S. Jin, A. Jinaru, O. Jinnouchi, M. D. Joergensen, P. Johansson, K. A. Johns, K. Jon-And, G. Jones, R. W. L. Jones, T. J. Jones, J. Jongmanns, P. M. Jorge, K. D. Joshi, J. Jovicevic, X. Ju, C. A. Jung, P. Jussel, A. Juste Rozas, M. Kaci, A. Kaczmarska, M. Kado, H. Kagan, M. Kagan, S. J. Kahn, E. Kajomovitz, C. W. Kalderon, S. Kama, A. Kamenshchikov, N. Kanaya, S. Kaneti, V. A. Kantserov, J. Kanzaki, B. Kaplan, L. S. Kaplan, A. Kapliy, D. Kar, K. Karakostas, A. Karamaoun, N. Karastathis, M. J. Kareem, E. Karentzos, M. Karnevskiy, S. N. Karpov, Z. M. Karpova, K. Karthik, V. Kartvelishvili, A. N. Karyukhin, L. Kashif, R. D. Kass, A. Kastanas, Y. Kataoka, C. Kato, A. Katre, J. Katzy, K. Kawagoe, T. Kawamoto, G. Kawamura, S. Kazama, V. F. Kazanin, R. Keeler, R. Kehoe, J. S. Keller, J. J. Kempster, H. Keoshkerian, O. Kepka, B. P. Kerševan, S. Kersten, R. A. Keyes, F. Khalil-zada, H. Khandanyan, A. Khanov, A. G. Kharlamov, T. J. Khoo, V. Khovanskiy, E. Khramov, J. Khubua, S. Kido, H. Y. Kim, S. H. Kim, Y. K. Kim, N. Kimura, O. M. Kind, B. T. King, M. King, S. B. King, J. Kirk, A. E. Kiryunin, T. Kishimoto, D. Kisielewska, F. Kiss, K. Kiuchi, O. Kivernyk, E. Kladiva, M. H. Klein, M. Klein, U. Klein, K. Kleinknecht, P. Klimek, A. Klimentov, R. Klingenberg, J. A. Klinger, T. Klioutchnikova, E.-E. Kluge, P. Kluit, S. Kluth, J. Knapik, E. Kneringer, E. B. F. G. Knoops, A. Knue, A. Kobayashi, D. Kobayashi, T. Kobayashi, M. Kobel, M. Kocian, P. Kodys, T. Koffas, E. Koffeman, L. A. Kogan, S. Kohlmann, Z. Kohout, T. Kohriki, T. Koi, H. Kolanoski, I. Koletsou, A. A. Komar, Y. Komori, T. Kondo, N. Kondrashova, K. Köneke, A. C. König, T. Kono, R. Konoplich, N. Konstantinidis, R. Kopeliansky, S. Koperny, L. Köpke, A. K. Kopp, K. Korcyl, K. Kordas, A. Korn, A. A. Korol, I. Korolkov, E. V. Korolkova, O. Kortner, S. Kortner, T. Kosek, V. V. Kostyukhin, V. M. Kotov, A. Kotwal, A. Kourkoumeli-Charalampidi, C. Kourkoumelis, V. Kouskoura, A. Koutsman, R. Kowalewski, T. Z. Kowalski, W. Kozanecki, A. S. Kozhin, V. A. Kramarenko, G. Kramberger, D. Krasnopevtsev, M. W. Krasny, A. Krasznahorkay, J. K. Kraus, A. Kravchenko, S. Kreiss, M. Kretz, J. Kretzschmar, K. Kreutzfeldt, P. Krieger, K. Krizka, K. Kroeninger, H. Kroha, J. Kroll, J. Kroseberg, J. Krstic, U. Kruchonak, H. Krüger, N. Krumnack, A. Kruse, M. C. Kruse, M. Kruskal, T. Kubota, H. Kucuk, S. Kuday, S. Kuehn, A. Kugel, F. Kuger, A. Kuhl, T. Kuhl, V. Kukhtin, Y. Kulchitsky, S. Kuleshov, M. Kuna, T. Kunigo, A. Kupco, H. Kurashige, Y. A. Kurochkin, V. Kus, E. S. Kuwertz, M. Kuze, J. Kvita, T. Kwan, D. Kyriazopoulos, A. La Rosa, J. L. La Rosa Navarro, L. La Rotonda, C. Lacasta, F. Lacava, J. Lacey, H. Lacker, D. Lacour, V. R. Lacuesta, E. Ladygin, R. Lafaye, B. Laforge, T. Lagouri, S. Lai, L. Lambourne, S. Lammers, C. L. Lampen, W. Lampl, E. Lançon, U. Landgraf, M. P. J. Landon, V. S. Lang, J. C. Lange, A. J. Lankford, F. Lanni, K. Lantzsch, A. Lanza, S. Laplace, C. Lapoire, J. F. Laporte, T. Lari, F. Lasagni Manghi, M. Lassnig, P. Laurelli, W. Lavrijsen, A. T. Law, P. Laycock, T. Lazovich, O. Le Dortz, E. Le Guirriec, E. Le Menedeu, M. LeBlanc, T. LeCompte, F. Ledroit-Guillon, C. A. Lee, S. C. Lee, L. Lee, G. Lefebvre, M. Lefebvre, F. Legger, C. Leggett, A. Lehan, G. Lehmann Miotto, X. Lei, W. A. Leight, A. Leisos, A. G. Leister, M. A. L. Leite, R. Leitner, D. Lellouch, B. Lemmer, K. J. C. Leney, T. Lenz, B. Lenzi, R. Leone, S. Leone, C. Leonidopoulos, S. Leontsinis, C. Leroy, C. G. Lester, M. Levchenko, J. Levêque, D. Levin, L. J. Levinson, M. Levy, A. Lewis, A. M. Leyko, M. Leyton, B. Li, H. Li, H. L. Li, L. Li, L. Li, S. Li, X. Li, Y. Li, Z. Liang, H. Liao, B. Liberti, A. Liblong, P. Lichard, K. Lie, J. Liebal, W. Liebig, C. Limbach, A. Limosani, S. C. Lin, T. H. Lin, F. Linde, B. E. Lindquist, J. T. Linnemann, E. Lipeles, A. Lipniacka, M. Lisovyi, T. M. Liss, D. Lissauer, A. Lister, A. M. Litke, B. Liu, D. Liu, H. Liu, J. Liu, J. B. Liu, K. Liu, L. Liu, M. Liu, M. Liu, Y. Liu, M. Livan, A. Lleres, J. Llorente Merino, S. L. Lloyd, F. Lo Sterzo, E. Lobodzinska, P. Loch, W. S. Lockman, F. K. Loebinger, A. E. Loevschall-Jensen, A. Loginov, T. Lohse, K. Lohwasser, M. Lokajicek, B. A. Long, J. D. Long, R. E. Long, K. A. Looper, L. Lopes, D. Lopez Mateos, B. Lopez Paredes, I. Lopez Paz, J. Lorenz, N. Lorenzo Martinez, M. Losada, P. Loscutoff, P. J. Lösel, X. Lou, A. Lounis, J. Love, P. A. Love, N. Lu, H. J. Lubatti, C. Luci, A. Lucotte, F. Luehring, W. Lukas, L. Luminari, O. Lundberg, B. Lund-Jensen, D. Lynn, R. Lysak, E. Lytken, H. Ma, L. L. Ma, G. Maccarrone, A. Macchiolo, C. M. Macdonald, B. Maček, J. Machado Miguens, D. Macina, D. Madaffari, R. Madar, H. J. Maddocks, W. F. Mader, A. Madsen, J. Maeda, S. Maeland, T. Maeno, A. Maevskiy, E. Magradze, K. Mahboubi, J. Mahlstedt, C. Maiani, C. Maidantchik, A. A. Maier, T. Maier, A. Maio, S. Majewski, Y. Makida, N. Makovec, B. Malaescu, Pa. Malecki, V. P. Maleev, F. Malek, U. Mallik, D. Malon, C. Malone, S. Maltezos, V. M. Malyshev, S. Malyukov, J. Mamuzic, G. Mancini, B. Mandelli, L. Mandelli, I. Mandić, R. Mandrysch, J. Maneira, A. Manfredini, L. Manhaes de Andrade Filho, J. Manjarres Ramos, A. Mann, A. Manousakis-Katsikakis, B. Mansoulie, R. Mantifel, M. Mantoani, L. Mapelli, L. March, G. Marchiori, M. Marcisovsky, C. P. Marino, M. Marjanovic, D. E. Marley, F. Marroquim, S. P. Marsden, Z. Marshall, L. F. Marti, S. Marti-Garcia, B. Martin, T. A. Martin, V. J. Martin, B. Martin dit Latour, M. Martinez, S. Martin-Haugh, V. S. Martoiu, A. C. Martyniuk, M. Marx, F. Marzano, A. Marzin, L. Masetti, T. Mashimo, R. Mashinistov, J. Masik, A. L. Maslennikov, I. Massa, L. Massa, N. Massol, P. Mastrandrea, A. Mastroberardino, T. Masubuchi, P. Mättig, J. Mattmann, J. Maurer, S. J. Maxfield, D. A. Maximov, R. Mazini, S. M. Mazza, L. Mazzaferro, G. Mc Goldrick, S. P. Mc Kee, A. McCarn, R. L. McCarthy, T. G. McCarthy, N. A. McCubbin, K. W. McFarlane, J. A. Mcfayden, G. Mchedlidze, S. J. McMahon, R. A. McPherson, M. Medinnis, S. Meehan, S. Mehlhase, A. Mehta, K. Meier, C. Meineck, B. Meirose, B. R. Mellado Garcia, F. Meloni, A. Mengarelli, S. Menke, E. Meoni, K. M. Mercurio, S. Mergelmeyer, P. Mermod, L. Merola, C. Meroni, F. S. Merritt, A. Messina, J. Metcalfe, A. S. Mete, C. Meyer, C. Meyer, J-P. Meyer, J. Meyer, H. Meyer Zu Theenhausen, R. P. Middleton, S. Miglioranzi, L. Mijović, G. Mikenberg, M. Mikestikova, M. Mikuž, M. Milesi, A. Milic, D. W. Miller, C. Mills, A. Milov, D. A. Milstead, A. A. Minaenko, Y. Minami, I. A. Minashvili, A. I. Mincer, B. Mindur, M. Mineev, Y. Ming, L. M. Mir, T. Mitani, J. Mitrevski, V. A. Mitsou, A. Miucci, P. S. Miyagawa, J. U. Mjörnmark, T. Moa, K. Mochizuki, S. Mohapatra, W. Mohr, S. Molander, R. Moles-Valls, K. Mönig, C. Monini, J. Monk, E. Monnier, J. Montejo Berlingen, F. Monticelli, S. Monzani, R. W. Moore, N. Morange, D. Moreno, M. Moreno Llácer, P. Morettini, D. Mori, M. Morii, M. Morinaga, V. Morisbak, S. Moritz, A. K. Morley, G. Mornacchi, J. D. Morris, S. S. Mortensen, A. Morton, L. Morvaj, M. Mosidze, J. Moss, K. Motohashi, R. Mount, E. Mountricha, S. V. Mouraviev, E. J. W. Moyse, S. Muanza, R. D. Mudd, F. Mueller, J. Mueller, R. S. P. Mueller, T. Mueller, D. Muenstermann, P. Mullen, G. A. Mullier, J. A. Murillo Quijada, W. J. Murray, H. Musheghyan, E. Musto, A. G. Myagkov, M. Myska, B. P. Nachman, O. Nackenhorst, J. Nadal, K. Nagai, R. Nagai, Y. Nagai, K. Nagano, A. Nagarkar, Y. Nagasaka, K. Nagata, M. Nagel, E. Nagy, A. M. Nairz, Y. Nakahama, K. Nakamura, T. Nakamura, I. Nakano, H. Namasivayam, R. F. Naranjo Garcia, R. Narayan, D. I. Narrias Villar, T. Naumann, G. Navarro, R. Nayyar, H. A. Neal, P. Yu. Nechaeva, T. J. Neep, P. D. Nef, A. Negri, M. Negrini, S. Nektarijevic, C. Nellist, A. Nelson, S. Nemecek, P. Nemethy, A. A. Nepomuceno, M. Nessi, M. S. Neubauer, M. Neumann, R. M. Neves, P. Nevski, P. R. Newman, D. H. Nguyen, R. B. Nickerson, R. Nicolaidou, B. Nicquevert, J. Nielsen, N. Nikiforou, A. Nikiforov, V. Nikolaenko, I. Nikolic-Audit, K. Nikolopoulos, J. K. Nilsen, P. Nilsson, Y. Ninomiya, A. Nisati, R. Nisius, T. Nobe, M. Nomachi, I. Nomidis, T. Nooney, S. Norberg, M. Nordberg, O. Novgorodova, S. Nowak, M. Nozaki, L. Nozka, K. Ntekas, G. Nunes Hanninger, T. Nunnemann, E. Nurse, F. Nuti, B. J. O’Brien, F. O’grady, D. C. O’Neil, V. O’Shea, F. G. Oakham, H. Oberlack, T. Obermann, J. Ocariz, A. Ochi, I. Ochoa, J. P. Ochoa-Ricoux, S. Oda, S. Odaka, H. Ogren, A. Oh, S. H. Oh, C. C. Ohm, H. Ohman, H. Oide, W. Okamura, H. Okawa, Y. Okumura, T. Okuyama, A. Olariu, S. A. Olivares Pino, D. Oliveira Damazio, E. Oliver Garcia, A. Olszewski, J. Olszowska, A. Onofre, P. U. E. Onyisi, C. J. Oram, M. J. Oreglia, Y. Oren, D. Orestano, N. Orlando, C. Oropeza Barrera, R. S. Orr, B. Osculati, R. Ospanov, G. Otero y Garzon, H. Otono, M. Ouchrif, F. Ould-Saada, A. Ouraou, K. P. Oussoren, Q. Ouyang, A. Ovcharova, M. Owen, R. E. Owen, V. E. Ozcan, N. Ozturk, K. Pachal, A. Pacheco Pages, C. Padilla Aranda, M. Pagáčová, S. Pagan Griso, E. Paganis, F. Paige, P. Pais, K. Pajchel, G. Palacino, S. Palestini, M. Palka, D. Pallin, A. Palma, Y. B. Pan, E. Panagiotopoulou, C. E. Pandini, J. G. Panduro Vazquez, P. Pani, S. Panitkin, D. Pantea, L. Paolozzi, Th. D. Papadopoulou, K. Papageorgiou, A. Paramonov, D. Paredes Hernandez, M. A. Parker, K. A. Parker, F. Parodi, J. A. Parsons, U. Parzefall, E. Pasqualucci, S. Passaggio, F. Pastore, Fr. Pastore, G. Pásztor, S. Pataraia, N. D. Patel, J. R. Pater, T. Pauly, J. Pearce, B. Pearson, L. E. Pedersen, M. Pedersen, S. Pedraza Lopez, R. Pedro, S. V. Peleganchuk, D. Pelikan, O. Penc, C. Peng, H. Peng, B. Penning, J. Penwell, D. V. Perepelitsa, E. Perez Codina, M. T. Pérez García-Estañ, L. Perini, H. Pernegger, S. Perrella, R. Peschke, V. D. Peshekhonov, K. Peters, R. F. Y. Peters, B. A. Petersen, T. C. Petersen, E. Petit, A. Petridis, C. Petridou, P. Petroff, E. Petrolo, F. Petrucci, N. E. Pettersson, R. Pezoa, P. W. Phillips, G. Piacquadio, E. Pianori, A. Picazio, E. Piccaro, M. Piccinini, M. A. Pickering, R. Piegaia, D. T. Pignotti, J. E. Pilcher, A. D. Pilkington, J. Pina, M. Pinamonti, J. L. Pinfold, A. Pingel, S. Pires, H. Pirumov, M. Pitt, C. Pizio, L. Plazak, M.-A. Pleier, V. Pleskot, E. Plotnikova, P. Plucinski, D. Pluth, R. Poettgen, L. Poggioli, D. Pohl, G. Polesello, A. Poley, A. Policicchio, R. Polifka, A. Polini, C. S. Pollard, V. Polychronakos, K. Pommès, L. Pontecorvo, B. G. Pope, G. A. Popeneciu, D. S. Popovic, A. Poppleton, S. Pospisil, K. Potamianos, I. N. Potrap, C. J. Potter, C. T. Potter, G. Poulard, J. Poveda, V. Pozdnyakov, P. Pralavorio, A. Pranko, S. Prasad, S. Prell, D. Price, L. E. Price, M. Primavera, S. Prince, M. Proissl, K. Prokofiev, F. Prokoshin, E. Protopapadaki, S. Protopopescu, J. Proudfoot, M. Przybycien, E. Ptacek, D. Puddu, E. Pueschel, D. Puldon, M. Purohit, P. Puzo, J. Qian, G. Qin, Y. Qin, A. Quadt, D. R. Quarrie, W. B. Quayle, M. Queitsch-Maitland, D. Quilty, S. Raddum, V. Radeka, V. Radescu, S. K. Radhakrishnan, P. Radloff, P. Rados, F. Ragusa, G. Rahal, S. Rajagopalan, M. Rammensee, C. Rangel-Smith, F. Rauscher, S. Rave, T. Ravenscroft, M. Raymond, A. L. Read, N. P. Readioff, D. M. Rebuzzi, A. Redelbach, G. Redlinger, R. Reece, K. Reeves, L. Rehnisch, J. Reichert, H. Reisin, M. Relich, C. Rembser, H. Ren, A. Renaud, M. Rescigno, S. Resconi, O. L. Rezanova, P. Reznicek, R. Rezvani, R. Richter, S. Richter, E. Richter-Was, O. Ricken, M. Ridel, P. Rieck, C. J. Riegel, J. Rieger, M. Rijssenbeek, A. Rimoldi, L. Rinaldi, B. Ristić, E. Ritsch, I. Riu, F. Rizatdinova, E. Rizvi, S. H. Robertson, A. Robichaud-Veronneau, D. Robinson, J. E. M. Robinson, A. Robson, C. Roda, S. Roe, O. Røhne, S. Rolli, A. Romaniouk, M. Romano, S. M. Romano Saez, E. Romero Adam, N. Rompotis, M. Ronzani, L. Roos, E. Ros, S. Rosati, K. Rosbach, P. Rose, P. L. Rosendahl, O. Rosenthal, V. Rossetti, E. Rossi, L. P. Rossi, J. H. N. Rosten, R. Rosten, M. Rotaru, I. Roth, J. Rothberg, D. Rousseau, C. R. Royon, A. Rozanov, Y. Rozen, X. Ruan, F. Rubbo, I. Rubinskiy, V. I. Rud, C. Rudolph, M. S. Rudolph, F. Rühr, A. Ruiz-Martinez, Z. Rurikova, N. A. Rusakovich, A. Ruschke, H. L. Russell, J. P. Rutherfoord, N. Ruthmann, Y. F. Ryabov, M. Rybar, G. Rybkin, N. C. Ryder, A. F. Saavedra, G. Sabato, S. Sacerdoti, A. Saddique, H. F-W. Sadrozinski, R. Sadykov, F. Safai Tehrani, M. Sahinsoy, M. Saimpert, T. Saito, H. Sakamoto, Y. Sakurai, G. Salamanna, A. Salamon, J. E. Salazar Loyola, M. Saleem, D. Salek, P. H. Sales De Bruin, D. Salihagic, A. Salnikov, J. Salt, D. Salvatore, F. Salvatore, A. Salvucci, A. Salzburger, D. Sammel, D. Sampsonidis, A. Sanchez, J. Sánchez, V. Sanchez Martinez, H. Sandaker, R. L. Sandbach, H. G. Sander, M. P. Sanders, M. Sandhoff, C. Sandoval, R. Sandstroem, D. P. C. Sankey, M. Sannino, A. Sansoni, C. Santoni, R. Santonico, H. Santos, I. Santoyo Castillo, K. Sapp, A. Sapronov, J. G. Saraiva, B. Sarrazin, O. Sasaki, Y. Sasaki, K. Sato, G. Sauvage, E. Sauvan, G. Savage, P. Savard, C. Sawyer, L. Sawyer, J. Saxon, C. Sbarra, A. Sbrizzi, T. Scanlon, D. A. Scannicchio, M. Scarcella, V. Scarfone, J. Schaarschmidt, P. Schacht, D. Schaefer, R. Schaefer, J. Schaeffer, S. Schaepe, S. Schaetzel, U. Schäfer, A. C. Schaffer, D. Schaile, R. D. Schamberger, V. Scharf, V. A. Schegelsky, D. Scheirich, M. Schernau, C. Schiavi, C. Schillo, M. Schioppa, S. Schlenker, K. Schmieden, C. Schmitt, S. Schmitt, S. Schmitt, B. Schneider, Y. J. Schnellbach, U. Schnoor, L. Schoeffel, A. Schoening, B. D. Schoenrock, E. Schopf, A. L. S. Schorlemmer, M. Schott, D. Schouten, J. Schovancova, S. Schramm, M. Schreyer, C. Schroeder, N. Schuh, M. J. Schultens, H.-C. Schultz-Coulon, H. Schulz, M. Schumacher, B. A. Schumm, Ph. Schune, C. Schwanenberger, A. Schwartzman, T. A. Schwarz, Ph. Schwegler, H. Schweiger, Ph. Schwemling, R. Schwienhorst, J. Schwindling, T. Schwindt, F. G. Sciacca, E. Scifo, G. Sciolla, F. Scuri, F. Scutti, J. Searcy, G. Sedov, E. Sedykh, P. Seema, S. C. Seidel, A. Seiden, F. Seifert, J. M. Seixas, G. Sekhniaidze, K. Sekhon, S. J. Sekula, D. M. Seliverstov, N. Semprini-Cesari, C. Serfon, L. Serin, L. Serkin, T. Serre, M. Sessa, R. Seuster, H. Severini, T. Sfiligoj, F. Sforza, A. Sfyrla, E. Shabalina, M. Shamim, L. Y. Shan, R. Shang, J. T. Shank, M. Shapiro, P. B. Shatalov, K. Shaw, S. M. Shaw, A. Shcherbakova, C. Y. Shehu, P. Sherwood, L. Shi, S. Shimizu, C. O. Shimmin, M. Shimojima, M. Shiyakova, A. Shmeleva, D. Shoaleh Saadi, M. J. Shochet, S. Shojaii, S. Shrestha, E. Shulga, M. A. Shupe, S. Shushkevich, P. Sicho, P. E. Sidebo, O. Sidiropoulou, D. Sidorov, A. Sidoti, F. Siegert, Dj. Sijacki, J. Silva, Y. Silver, S. B. Silverstein, V. Simak, O. Simard, Lj. Simic, S. Simion, E. Simioni, B. Simmons, D. Simon, P. Sinervo, N. B. Sinev, M. Sioli, G. Siragusa, A. N. Sisakyan, S. Yu. Sivoklokov, J. Sjölin, T. B. Sjursen, M. B. Skinner, H. P. Skottowe, P. Skubic, M. Slater, T. Slavicek, M. Slawinska, K. Sliwa, V. Smakhtin, B. H. Smart, L. Smestad, S. Yu. Smirnov, Y. Smirnov, L. N. Smirnova, O. Smirnova, M. N. K. Smith, R. W. Smith, M. Smizanska, K. Smolek, A. A. Snesarev, G. Snidero, S. Snyder, R. Sobie, F. Socher, A. Soffer, D. A. Soh, G. Sokhrannyi, C. A. Solans, M. Solar, J. Solc, E. Yu. Soldatov, U. Soldevila, A. A. Solodkov, A. Soloshenko, O. V. Solovyanov, V. Solovyev, P. Sommer, H. Y. Song, N. Soni, A. Sood, A. Sopczak, B. Sopko, V. Sopko, V. Sorin, D. Sosa, M. Sosebee, C. L. Sotiropoulou, R. Soualah, A. M. Soukharev, D. South, B. C. Sowden, S. Spagnolo, M. Spalla, M. Spangenberg, F. Spanò, W. R. Spearman, D. Sperlich, F. Spettel, R. Spighi, G. Spigo, L. A. Spiller, M. Spousta, T. Spreitzer, R. D. St. Denis, S. Staerz, J. Stahlman, R. Stamen, S. Stamm, E. Stanecka, C. Stanescu, M. Stanescu-Bellu, M. M. Stanitzki, S. Stapnes, E. A. Starchenko, J. Stark, P. Staroba, P. Starovoitov, R. Staszewski, P. Stavina, P. Steinberg, B. Stelzer, H. J. Stelzer, O. Stelzer-Chilton, H. Stenzel, G. A. Stewart, J. A. Stillings, M. C. Stockton, M. Stoebe, G. Stoicea, P. Stolte, S. Stonjek, A. R. Stradling, A. Straessner, M. E. Stramaglia, J. Strandberg, S. Strandberg, A. Strandlie, E. Strauss, M. Strauss, P. Strizenec, R. Ströhmer, D. M. Strom, R. Stroynowski, A. Strubig, S. A. Stucci, B. Stugu, N. A. Styles, D. Su, J. Su, R. Subramaniam, A. Succurro, Y. Sugaya, C. Suhr, M. Suk, V. V. Sulin, S. Sultansoy, T. Sumida, S. Sun, X. Sun, J. E. Sundermann, K. Suruliz, G. Susinno, M. R. Sutton, S. Suzuki, M. Svatos, M. Swiatlowski, I. Sykora, T. Sykora, D. Ta, C. Taccini, K. Tackmann, J. Taenzer, A. Taffard, R. Tafirout, N. Taiblum, H. Takai, R. Takashima, H. Takeda, T. Takeshita, Y. Takubo, M. Talby, A. A. Talyshev, J. Y. C. Tam, K. G. Tan, J. Tanaka, R. Tanaka, S. Tanaka, B. B. Tannenwald, N. Tannoury, S. Tapprogge, S. Tarem, F. Tarrade, G. F. Tartarelli, P. Tas, M. Tasevsky, T. Tashiro, E. Tassi, A. Tavares Delgado, Y. Tayalati, F. E. Taylor, G. N. Taylor, W. Taylor, F. A. Teischinger, M. Teixeira Dias Castanheira, P. Teixeira-Dias, K. K. Temming, D. Temple, H. Ten Kate, P. K. Teng, J. J. Teoh, F. Tepel, S. Terada, K. Terashi, J. Terron, S. Terzo, M. Testa, R. J. Teuscher, T. Theveneaux-Pelzer, J. P. Thomas, J. Thomas-Wilsker, E. N. Thompson, P. D. Thompson, R. J. Thompson, A. S. Thompson, L. A. Thomsen, E. Thomson, M. Thomson, R. P. Thun, M. J. Tibbetts, R. E. Ticse Torres, V. O. Tikhomirov, Yu. A. Tikhonov, S. Timoshenko, E. Tiouchichine, P. Tipton, S. Tisserant, K. Todome, T. Todorov, S. Todorova-Nova, J. Tojo, S. Tokár, K. Tokushuku, K. Tollefson, E. Tolley, L. Tomlinson, M. Tomoto, L. Tompkins, K. Toms, E. Torrence, H. Torres, E. Torró Pastor, J. Toth, F. Touchard, D. R. Tovey, T. Trefzger, L. Tremblet, A. Tricoli, I. M. Trigger, S. Trincaz-Duvoid, M. F. Tripiana, W. Trischuk, B. Trocmé, C. Troncon, M. Trottier-McDonald, M. Trovatelli, P. True, L. Truong, M. Trzebinski, A. Trzupek, C. Tsarouchas, J. C-L. Tseng, P. V. Tsiareshka, D. Tsionou, G. Tsipolitis, N. Tsirintanis, S. Tsiskaridze, V. Tsiskaridze, E. G. Tskhadadze, I. I. Tsukerman, V. Tsulaia, S. Tsuno, D. Tsybychev, A. Tudorache, V. Tudorache, A. N. Tuna, S. A. Tupputi, S. Turchikhin, D. Turecek, R. Turra, A. J. Turvey, P. M. Tuts, A. Tykhonov, M. Tylmad, M. Tyndel, I. Ueda, R. Ueno, M. Ughetto, M. Ugland, F. Ukegawa, G. Unal, A. Undrus, G. Unel, F. C. Ungaro, Y. Unno, C. Unverdorben, J. Urban, P. Urquijo, P. Urrejola, G. Usai, A. Usanova, L. Vacavant, V. Vacek, B. Vachon, C. Valderanis, N. Valencic, S. Valentinetti, A. Valero, L. Valery, S. Valkar, E. Valladolid Gallego, S. Vallecorsa, J. A. Valls Ferrer, W. Van Den Wollenberg, P. C. Van Der Deijl, R. van der Geer, H. van der Graaf, N. van Eldik, P. van Gemmeren, J. Van Nieuwkoop, I. van Vulpen, M. C. van Woerden, M. Vanadia, W. Vandelli, R. Vanguri, A. Vaniachine, F. Vannucci, G. Vardanyan, R. Vari, E. W. Varnes, T. Varol, D. Varouchas, A. Vartapetian, K. E. Varvell, F. Vazeille, T. Vazquez Schroeder, J. Veatch, L. M. Veloce, F. Veloso, T. Velz, S. Veneziano, A. Ventura, D. Ventura, M. Venturi, N. Venturi, A. Venturini, V. Vercesi, M. Verducci, W. Verkerke, J. C. Vermeulen, A. Vest, M. C. Vetterli, O. Viazlo, I. Vichou, T. Vickey, O. E. Vickey Boeriu, G. H. A. Viehhauser, S. Viel, R. Vigne, M. Villa, M. Villaplana Perez, E. Vilucchi, M. G. Vincter, V. B. Vinogradov, I. Vivarelli, F. Vives Vaque, S. Vlachos, D. Vladoiu, M. Vlasak, M. Vogel, P. Vokac, G. Volpi, M. Volpi, H. von der Schmitt, H. von Radziewski, E. von Toerne, V. Vorobel, K. Vorobev, M. Vos, R. Voss, J. H. Vossebeld, N. Vranjes, M. Vranjes Milosavljevic, V. Vrba, M. Vreeswijk, R. Vuillermet, I. Vukotic, Z. Vykydal, P. Wagner, W. Wagner, H. Wahlberg, S. Wahrmund, J. Wakabayashi, J. Walder, R. Walker, W. Walkowiak, C. Wang, F. Wang, H. Wang, H. Wang, J. Wang, J. Wang, K. Wang, R. Wang, S. M. Wang, T. Wang, T. Wang, X. Wang, C. Wanotayaroj, A. Warburton, C. P. Ward, D. R. Wardrope, A. Washbrook, C. Wasicki, P. M. Watkins, A. T. Watson, I. J. Watson, M. F. Watson, G. Watts, S. Watts, B. M. Waugh, S. Webb, M. S. Weber, S. W. Weber, J. S. Webster, A. R. Weidberg, B. Weinert, J. Weingarten, C. Weiser, H. Weits, P. S. Wells, T. Wenaus, T. Wengler, S. Wenig, N. Wermes, M. Werner, P. Werner, M. Wessels, J. Wetter, K. Whalen, A. M. Wharton, A. White, M. J. White, R. White, S. White, D. Whiteson, F. J. Wickens, W. Wiedenmann, M. Wielers, P. Wienemann, C. Wiglesworth, L. A. M. Wiik-Fuchs, A. Wildauer, H. G. Wilkens, H. H. Williams, S. Williams, C. Willis, S. Willocq, A. Wilson, J. A. Wilson, I. Wingerter-Seez, F. Winklmeier, B. T. Winter, M. Wittgen, J. Wittkowski, S. J. Wollstadt, M. W. Wolter, H. Wolters, B. K. Wosiek, J. Wotschack, M. J. Woudstra, K. W. Wozniak, M. Wu, M. Wu, S. L. Wu, X. Wu, Y. Wu, T. R. Wyatt, B. M. Wynne, S. Xella, D. Xu, L. Xu, B. Yabsley, S. Yacoob, R. Yakabe, M. Yamada, D. Yamaguchi, Y. Yamaguchi, A. Yamamoto, S. Yamamoto, T. Yamanaka, K. Yamauchi, Y. Yamazaki, Z. Yan, H. Yang, H. Yang, Y. Yang, W-M. Yao, Y. Yasu, E. Yatsenko, K. H. Yau Wong, J. Ye, S. Ye, I. Yeletskikh, A. L. Yen, E. Yildirim, K. Yorita, R. Yoshida, K. Yoshihara, C. Young, C. J. S. Young, S. Youssef, D. R. Yu, J. Yu, J. M. Yu, J. Yu, L. Yuan, S. P. Y. Yuen, A. Yurkewicz, I. Yusuff, B. Zabinski, R. Zaidan, A. M. Zaitsev, J. Zalieckas, A. Zaman, S. Zambito, L. Zanello, D. Zanzi, C. Zeitnitz, M. Zeman, A. Zemla, Q. Zeng, K. Zengel, O. Zenin, T. Ženiš, D. Zerwas, D. Zhang, F. Zhang, H. Zhang, J. Zhang, L. Zhang, R. Zhang, X. Zhang, Z. Zhang, X. Zhao, Y. Zhao, Z. Zhao, A. Zhemchugov, J. Zhong, B. Zhou, C. Zhou, L. Zhou, L. Zhou, M. Zhou, N. Zhou, C. G. Zhu, H. Zhu, J. Zhu, Y. Zhu, X. Zhuang, K. Zhukov, A. Zibell, D. Zieminska, N. I. Zimine, C. Zimmermann, S. Zimmermann, Z. Zinonos, M. Zinser, M. Ziolkowski, L. Živković, G. Zobernig, A. Zoccoli, M. zur Nedden, G. Zurzolo, L. Zwalinski, ATLAS Collaboration

**Affiliations:** Department of Physics, University of Adelaide, Adelaide, Australia; Physics Department, SUNY Albany, Albany, NY USA; Department of Physics, University of Alberta, Edmonton, AB Canada; Department of Physics, Ankara University, Ankara, Turkey; Istanbul Aydin University, Istanbul, Turkey; Division of Physics, TOBB University of Economics and Technology, Ankara, Turkey; LAPP, CNRS/IN2P3 and Université Savoie Mont Blanc, Annecy-le-Vieux, France; High Energy Physics Division, Argonne National Laboratory, Argonne, IL USA; Department of Physics, University of Arizona, Tucson, AZ USA; Department of Physics, The University of Texas at Arlington, Arlington, TX USA; Physics Department, University of Athens, Athens, Greece; Physics Department, National Technical University of Athens, Zografou, Greece; Institute of Physics, Azerbaijan Academy of Sciences, Baku, Azerbaijan; Institut de Física d’Altes Energies and Departament de Física de la Universitat Autònoma de Barcelona, Barcelona, Spain; Institute of Physics, University of Belgrade, Belgrade, Serbia; Department for Physics and Technology, University of Bergen, Bergen, Norway; Physics Division, Lawrence Berkeley National Laboratory and University of California, Berkeley, CA USA; Department of Physics, Humboldt University, Berlin, Germany; Albert Einstein Center for Fundamental Physics and Laboratory for High Energy Physics, University of Bern, Bern, Switzerland; School of Physics and Astronomy, University of Birmingham, Birmingham, UK; Department of Physics, Bogazici University, Istanbul, Turkey; Department of Physics Engineering, Gaziantep University, Gaziantep, Turkey; Department of Physics, Dogus University, Istanbul, Turkey; INFN Sezione di Bologna, Bologna, Italy; Dipartimento di Fisica e Astronomia, Università di Bologna, Bologna, Italy; Physikalisches Institut, University of Bonn, Bonn, Germany; Department of Physics, Boston University, Boston, MA USA; Department of Physics, Brandeis University, Waltham, MA USA; Universidade Federal do Rio De Janeiro COPPE/EE/IF, Rio de Janeiro, Brazil; Electrical Circuits Department, Federal University of Juiz de Fora (UFJF), Juiz de Fora, Brazil; Federal University of Sao Joao del Rei (UFSJ), Sao Joao del Rei, Brazil; Instituto de Fisica, Universidade de Sao Paulo, Sao Paulo, Brazil; Physics Department, Brookhaven National Laboratory, Upton, NY USA; National Institute of Physics and Nuclear Engineering, Bucharest, Romania; Physics Department, National Institute for Research and Development of Isotopic and Molecular Technologies, Cluj Napoca, Romania; University Politehnica Bucharest, Bucharest, Romania; West University in Timisoara, Timisoara, Romania; Departamento de Física, Universidad de Buenos Aires, Buenos Aires, Argentina; Cavendish Laboratory, University of Cambridge, Cambridge, UK; Department of Physics, Carleton University, Ottawa, ON Canada; CERN, Geneva, Switzerland; Enrico Fermi Institute, University of Chicago, Chicago, IL USA; Departamento de Física, Pontificia Universidad Católica de Chile, Santiago, Chile; Departamento de Física, Universidad Técnica Federico Santa María, Valparaiso, Chile; Institute of High Energy Physics, Chinese Academy of Sciences, Beijing, China; Department of Modern Physics, University of Science and Technology of China, Anhui, China; Department of Physics, Nanjing University, Jiangsu, China; School of Physics, Shandong University, Shandong, China; Shanghai Key Laboratory for Particle Physics and Cosmology, Department of Physics and Astronomy, Shanghai Jiao Tong University, Shanghai, China; Physics Department, Tsinghua University, Beijing, 100084 China; Laboratoire de Physique Corpusculaire, Clermont Université and Université Blaise Pascal and CNRS/IN2P3, Clermont-Ferrand, France; Nevis Laboratory, Columbia University, Irvington, NY USA; Niels Bohr Institute, University of Copenhagen, Kobenhavn, Denmark; INFN Gruppo Collegato di Cosenza, Laboratori Nazionali di Frascati, Frascati, Italy; Dipartimento di Fisica, Università della Calabria, Rende, Italy; AGH University of Science and Technology, Faculty of Physics and Applied Computer Science, Krakow, Poland; Marian Smoluchowski Institute of Physics, Jagiellonian University, Krakow, Poland; Institute of Nuclear Physics, Polish Academy of Sciences, Krakow, Poland; Physics Department, Southern Methodist University, Dallas, TX USA; Physics Department, University of Texas at Dallas, Richardson, TX USA; DESY, Hamburg and Zeuthen, Germany; Institut für Experimentelle Physik IV, Technische Universität Dortmund, Dortmund, Germany; Institut für Kern- und Teilchenphysik, Technische Universität Dresden, Dresden, Germany; Department of Physics, Duke University, Durham, NC USA; SUPA-School of Physics and Astronomy, University of Edinburgh, Edinburgh, UK; INFN Laboratori Nazionali di Frascati, Frascati, Italy; Fakultät für Mathematik und Physik, Albert-Ludwigs-Universität, Freiburg, Germany; Section de Physique, Université de Genève, Geneva, Switzerland; INFN Sezione di Genova, Genoa, Italy; Dipartimento di Fisica, Università di Genova, Genoa, Italy; E. Andronikashvili Institute of Physics, Iv. Javakhishvili Tbilisi State University, Tbilisi, Georgia; High Energy Physics Institute, Tbilisi State University, Tbilisi, Georgia; II Physikalisches Institut, Justus-Liebig-Universität Giessen, Giessen, Germany; SUPA-School of Physics and Astronomy, University of Glasgow, Glasgow, UK; II Physikalisches Institut, Georg-August-Universität, Göttingen, Germany; Laboratoire de Physique Subatomique et de Cosmologie, Université Grenoble-Alpes, CNRS/IN2P3, Grenoble, France; Department of Physics, Hampton University, Hampton, VA USA; Laboratory for Particle Physics and Cosmology, Harvard University, Cambridge, MA USA; Kirchhoff-Institut für Physik, Ruprecht-Karls-Universität Heidelberg, Heidelberg, Germany; Physikalisches Institut, Ruprecht-Karls-Universität Heidelberg, Heidelberg, Germany; ZITI Institut für technische Informatik, Ruprecht-Karls-Universität Heidelberg, Mannheim, Germany; Faculty of Applied Information Science, Hiroshima Institute of Technology, Hiroshima, Japan; Department of Physics, The Chinese University of Hong Kong, Shatin, N.T. Hong Kong; Department of Physics, The University of Hong Kong, Pokfulam, Hong Kong; Department of Physics, The Hong Kong University of Science and Technology, Clear Water Bay, Kowloon, Hong Kong, China; Department of Physics, Indiana University, Bloomington, IN USA; Institut für Astro- und Teilchenphysik, Leopold-Franzens-Universität, Innsbruck, Austria; University of Iowa, Iowa City, IA USA; Department of Physics and Astronomy, Iowa State University, Ames, IA USA; Joint Institute for Nuclear Research, JINR Dubna, Dubna, Russia; KEK, High Energy Accelerator Research Organization, Tsukuba, Japan; Graduate School of Science, Kobe University, Kobe, Japan; Faculty of Science, Kyoto University, Kyoto, Japan; Kyoto University of Education, Kyoto, Japan; Department of Physics, Kyushu University, Fukuoka, Japan; Instituto de Física La Plata, Universidad Nacional de La Plata and CONICET, La Plata, Argentina; Physics Department, Lancaster University, Lancaster, UK; INFN Sezione di Lecce, Lecce, Italy; Dipartimento di Matematica e Fisica, Università del Salento, Lecce, Italy; Oliver Lodge Laboratory, University of Liverpool, Liverpool, UK; Department of Physics, Jožef Stefan Institute and University of Ljubljana, Ljubljana, Slovenia; School of Physics and Astronomy, Queen Mary University of London, London, UK; Department of Physics, Royal Holloway University of London, Surrey, UK; Department of Physics and Astronomy, University College London, London, UK; Louisiana Tech University, Ruston, LA USA; Laboratoire de Physique Nucléaire et de Hautes Energies, UPMC and Université Paris-Diderot and CNRS/IN2P3, Paris, France; Fysiska institutionen, Lunds universitet, Lund, Sweden; Departamento de Fisica Teorica C-15, Universidad Autonoma de Madrid, Madrid, Spain; Institut für Physik, Universität Mainz, Mainz, Germany; School of Physics and Astronomy, University of Manchester, Manchester, UK; CPPM, Aix-Marseille Université and CNRS/IN2P3, Marseille, France; Department of Physics, University of Massachusetts, Amherst, MA USA; Department of Physics, McGill University, Montreal, QC Canada; School of Physics, University of Melbourne, Melbourne, Victoria Australia; Department of Physics, The University of Michigan, Ann Arbor, MI USA; Department of Physics and Astronomy, Michigan State University, East Lansing, MI USA; INFN Sezione di Milano, Milan, Italy; Dipartimento di Fisica, Università di Milano, Milan, Italy; B.I. Stepanov Institute of Physics, National Academy of Sciences of Belarus, Minsk, Republic of Belarus; National Scientific and Educational Centre for Particle and High Energy Physics, Minsk, Republic of Belarus; Department of Physics, Massachusetts Institute of Technology, Cambridge, MA USA; Group of Particle Physics, University of Montreal, Montreal, QC Canada; P.N. Lebedev Institute of Physics, Academy of Sciences, Moscow, Russia; Institute for Theoretical and Experimental Physics (ITEP), Moscow, Russia; National Research Nuclear University MEPhI, Moscow, Russia; D.V. Skobeltsyn Institute of Nuclear Physics, M.V. Lomonosov Moscow State University, Moscow, Russia; Fakultät für Physik, Ludwig-Maximilians-Universität München, München, Germany; Max-Planck-Institut für Physik (Werner-Heisenberg-Institut), München, Germany; Nagasaki Institute of Applied Science, Nagasaki, Japan; Graduate School of Science and Kobayashi-Maskawa Institute, Nagoya University, Nagoya, Japan; INFN Sezione di Napoli, Naples, Italy; Dipartimento di Fisica, Università di Napoli, Naples, Italy; Department of Physics and Astronomy, University of New Mexico, Albuquerque, NM USA; Institute for Mathematics, Astrophysics and Particle Physics, Radboud University Nijmegen/Nikhef, Nijmegen, The Netherlands; Nikhef National Institute for Subatomic Physics and University of Amsterdam, Amsterdam, The Netherlands; Department of Physics, Northern Illinois University, De Kalb, IL USA; Budker Institute of Nuclear Physics, SB RAS, Novosibirsk, Russia; Department of Physics, New York University, New York, NY USA; Ohio State University, Columbus, OH USA; Faculty of Science, Okayama University, Okayama, Japan; Homer L. Dodge Department of Physics and Astronomy, University of Oklahoma, Norman, OK USA; Department of Physics, Oklahoma State University, Stillwater, OK USA; Palacký University, RCPTM, Olomouc, Czech Republic; Center for High Energy Physics, University of Oregon, Eugene, OR USA; LAL, Université Paris-Sud and CNRS/IN2P3, Orsay, France; Graduate School of Science, Osaka University, Osaka, Japan; Department of Physics, University of Oslo, Oslo, Norway; Department of Physics, Oxford University, Oxford, UK; INFN Sezione di Pavia, Pavia, Italy; Dipartimento di Fisica, Università di Pavia, Pavia, Italy; Department of Physics, University of Pennsylvania, Philadelphia, PA USA; National Research Centre ”Kurchatov Institute” B.P.Konstantinov Petersburg Nuclear Physics Institute, St. Petersburg, Russia; INFN Sezione di Pisa, Pisa, Italy; Dipartimento di Fisica E. Fermi, Università di Pisa, Pisa, Italy; Department of Physics and Astronomy, University of Pittsburgh, Pittsburgh, PA USA; Laboratório de Instrumentação e Física Experimental de Partículas-LIP, Lisbon, Portugal; Faculdade de Ciências, Universidade de Lisboa, Lisbon, Portugal; Department of Physics, University of Coimbra, Coimbra, Portugal; Centro de Física Nuclear da Universidade de Lisboa, Lisbon, Portugal; Departamento de Fisica, Universidade do Minho, Braga, Portugal; Departamento de Fisica Teorica y del Cosmos and CAFPE, Universidad de Granada, Granada, Spain; Dep Fisica and CEFITEC of Faculdade de Ciencias e Tecnologia, Universidade Nova de Lisboa, Caparica, Portugal; Institute of Physics, Academy of Sciences of the Czech Republic, Praha, Czech Republic; Czech Technical University in Prague, Praha, Czech Republic; Faculty of Mathematics and Physics, Charles University in Prague, Prague, Czech Republic; State Research Center Institute for High Energy Physics, Protvino, Russia; Particle Physics Department, Rutherford Appleton Laboratory, Didcot, UK; INFN Sezione di Roma, Roma, Italy; Dipartimento di Fisica, Sapienza Università di Roma, Roma, Italy; INFN Sezione di Roma Tor Vergata, Rome, Italy; Dipartimento di Fisica, Università di Roma Tor Vergata, Roma, Italy; INFN Sezione di Roma Tre, Rome, Italy; Dipartimento di Matematica e Fisica, Università Roma Tre, Roma, Italy; Faculté des Sciences Ain Chock, Réseau Universitaire de Physique des Hautes Energies-Université Hassan II, Casablanca, Morocco; Centre National de l’Energie des Sciences Techniques Nucleaires, Rabat, Morocco; Faculté des Sciences Semlalia, Université Cadi Ayyad, LPHEA-Marrakech, Marrakech, Morocco; Faculté des Sciences, Université Mohamed Premier and LPTPM, Oujda, Morocco; Faculté des Sciences, Université Mohammed V, Rabat, Morocco; DSM/IRFU (Institut de Recherches sur les Lois Fondamentales de l’Univers), CEA Saclay (Commissariat à l’Energie Atomique et aux Energies Alternatives), Gif-sur-Yvette, France; Santa Cruz Institute for Particle Physics, University of California Santa Cruz, Santa Cruz, CA USA; Department of Physics, University of Washington, Seattle, WA USA; Department of Physics and Astronomy, University of Sheffield, Sheffield, UK; Department of Physics, Shinshu University, Nagano, Japan; Fachbereich Physik, Universität Siegen, Siegen, Germany; Department of Physics, Simon Fraser University, Burnaby, BC Canada; SLAC National Accelerator Laboratory, Stanford, CA USA; Faculty of Mathematics, Physics and Informatics, Comenius University, Bratislava, Slovak Republic; Department of Subnuclear Physics, Institute of Experimental Physics of the Slovak Academy of Sciences, Kosice, Slovak Republic; Department of Physics, University of Cape Town, Cape Town, South Africa; Department of Physics, University of Johannesburg, Johannesburg, South Africa; School of Physics, University of the Witwatersrand, Johannesburg, South Africa; Department of Physics, Stockholm University, Stockholm, Sweden; The Oskar Klein Centre, Stockholm, Sweden; Physics Department, Royal Institute of Technology, Stockholm, Sweden; Departments of Physics and Astronomy and Chemistry, Stony Brook University, Stony Brook, NY USA; Department of Physics and Astronomy, University of Sussex, Brighton, UK; School of Physics, University of Sydney, Sydney, Australia; Institute of Physics, Academia Sinica, Taipei, Taiwan; Department of Physics, Technion: Israel Institute of Technology, Haifa, Israel; Raymond and Beverly Sackler School of Physics and Astronomy, Tel Aviv University, Tel Aviv, Israel; Department of Physics, Aristotle University of Thessaloniki, Thessaloníki, Greece; International Center for Elementary Particle Physics and Department of Physics, The University of Tokyo, Tokyo, Japan; Graduate School of Science and Technology, Tokyo Metropolitan University, Tokyo, Japan; Department of Physics, Tokyo Institute of Technology, Tokyo, Japan; Department of Physics, University of Toronto, Toronto, ON Canada; TRIUMF, Vancouver, BC Canada; Department of Physics and Astronomy, York University, Toronto, ON Canada; Faculty of Pure and Applied Sciences, University of Tsukuba, Tsukuba, Japan; Department of Physics and Astronomy, Tufts University, Medford, MA USA; Centro de Investigaciones, Universidad Antonio Narino, Bogotá, Colombia; Department of Physics and Astronomy, University of California Irvine, Irvine, CA USA; INFN Gruppo Collegato di Udine, Sezione di Trieste, Udine, Italy; ICTP, Trieste, Italy; Dipartimento di Chimica Fisica e Ambiente, Università di Udine, Udine, Italy; Department of Physics, University of Illinois, Urbana, IL USA; Department of Physics and Astronomy, University of Uppsala, Uppsala, Sweden; Instituto de Física Corpuscular (IFIC) and Departamento de Física Atómica, Molecular y Nuclear and Departamento de Ingeniería Electrónica and Instituto de Microelectrónica de Barcelona (IMB-CNM), University of Valencia and CSIC, Valencia, Spain; Department of Physics, University of British Columbia, Vancouver, BC Canada; Department of Physics and Astronomy, University of Victoria, Victoria, BC Canada; Department of Physics, University of Warwick, Coventry, UK; Waseda University, Tokyo, Japan; Department of Particle Physics, The Weizmann Institute of Science, Rehovot, Israel; Department of Physics, University of Wisconsin, Madison, WI USA; Fakultät für Physik und Astronomie, Julius-Maximilians-Universität, Würzburg, Germany; Fachbereich C Physik, Bergische Universität Wuppertal, Wuppertal, Germany; Department of Physics, Yale University, New Haven, CT USA; Yerevan Physics Institute, Yerevan, Armenia; Centre de Calcul de l’Institut National de Physique Nucléaire et de Physique des Particules (IN2P3), Villeurbanne, France; CERN, 1211 Geneva 23, Switzerland

## Abstract

A search for the flavour-changing neutral-current decay $t\rightarrow qZ$ is presented. Data collected by the ATLAS detector during 2012 from proton–proton collisions at the Large Hadron Collider at a centre-of-mass energy of $\sqrt{s}=8$ TeV, corresponding to an integrated luminosity of 20.3 fb$^{-1}$, are analysed. Top-quark pair-production events with one top quark decaying through the $t\rightarrow qZ$ ($q=u,c$) channel and the other through the dominant Standard Model mode $t\rightarrow bW$ are considered as signal. Only the decays of the *Z* boson to charged leptons and leptonic *W* boson decays are used. No evidence for a signal is found and an observed (expected) upper limit on the $t\rightarrow qZ$ branching ratio of $7\times 10^{-4}$ ($8\times 10^{-4}$) is set at the 95 % confidence level

## Introduction

The top quark is the heaviest elementary particle known, with a mass $m_t=173.21\pm 0.51({\mathrm {stat.}})\pm 0.71({\mathrm {syst.}})$ GeV [1]. Its lifetime is so short that, within the Standard Model (SM) of particle physics, it decays (almost exclusively to *bW*) before hadronisation occurs. These properties make it a particle well suited to test the predictions of the SM. In the SM, due to the GIM mechanism [2], flavour-changing neutral current (FCNC) decays such as $t\rightarrow qZ$ are forbidden at tree level. They are allowed at one-loop level, but with a suppression factor of several orders of magnitude with respect to the dominant decay mode [3]. However, several SM extensions predict higher branching ratios (BRs) for the top-quark FCNC decays. Examples of such extensions are the quark-singlet model (QS) [4], the two-Higgs-doublet model with (FC 2HDM) or without (2HDM) flavour conservation [5], the minimal supersymmetric model (MSSM) [6], supersymmetry with R-parity violation (  SUSY) [7] or models with warped extra dimensions (RS) [8]. For a review see Ref. [9]. The maximum values for the $t\rightarrow qZ$ BRs predicted by these models and by the SM are summarised in Table 1. Experimental limits on the FCNC $t\rightarrow qZ$ BR were established by experiments at the Large Electron Positron Collider (LEP) [10–14], HERA [15], Tevatron [16, 17] and Large Hadron Collider (LHC) [18, 19]. The most stringent limit, BR$(t\rightarrow qZ) < 5\times 10^{-4}$, is the one from the CMS Collaboration [19] using 25 fb$^{-1}$ of data collected at $\sqrt{s}=7$ TeV and $\sqrt{s}=8$ TeV. Previous ATLAS results obtained at $\sqrt{s}=7$ TeV are also reported [18]. Limits on other FCNC top-quark decay BRs ($t\rightarrow qX, X=\gamma ,g,H$) are reported in Refs. [10–14, 20–28].

This paper presents the ATLAS results from the search for the FCNC decay $t\rightarrow qZ$ in $t\bar{t}$ events produced at $\sqrt{s}=8$ TeV, with one top quark decaying through the FCNC mode and the other through the SM dominant mode ($t\rightarrow bW$). Only the decays of the *Z* boson to charged leptons and leptonic *W* boson decays are considered. The final-state topology is thus characterised by the presence of three isolated charged leptons, at least two jets, and missing transverse momentum from the undetected neutrino. The paper is organised as follows. A brief description of the ATLAS detector is given in Sect. 2. The collected data samples and the simulations of signal and SM background processes are described in Sect. 3. Section 4 presents the object definitions, while the event analysis and kinematic reconstruction are explained in Sect. 5. Background evaluation and sources of systematic uncertainty are described in Sects. 6 and 7. Results are presented in Sect. 8 and conclusions are drawn in Sect. 9.

## Detector and data samples

The ATLAS experiment is a multi-purpose particle physics detector consisting of several sub-detector systems, which cover almost fully the solid angle^1^ around the interaction point. It is composed of an inner tracking system close to the interaction point and immersed in a 2 T axial magnetic field produced by a thin superconducting solenoid, a lead/liquid-argon (LAr) electromagnetic calorimeter, an iron/scintillator-tile hadronic calorimeter, copper/LAr hadronic endcap calorimeter and a muon spectrometer with three superconducting magnets, each one with eight toroid coils. The forward region is covered by additional LAr calorimeters with copper and tungsten absorbers. The combination of all these systems provides charged-particle momentum measurements, together with efficient and precise lepton and photon identification in the pseudorapidity range $|\eta | < 2.5$. Energy deposits over the full coverage of the calorimeters, $|\eta | < 4.9$ are used to reconstruct jets and missing transverse momentum (with magnitude $E_{\text {T}}^{\text {miss}}$). A three-level trigger system is used to select interesting events. The Level-1 trigger is implemented in hardware and uses a subset of detector information to reduce the event rate to a design value of at most 75 kHz. This is followed by two software-based trigger levels which together reduce the event rate to less than 1 kHz. A detailed description of the ATLAS detector is provided in Ref. [29].

In this paper the full 2012 dataset from proton–proton (*pp*) collisions at $\sqrt{s}=8$ TeV is used. The analysed events were recorded by single-electron or single-muon triggers and fulfil standard data-quality requirements. Triggers with different transverse momentum thresholds are used to increase the overall efficiency. The triggers using a low transverse momentum ($p_{\text {T}}$) threshold ($p_{\text {T}} ^{e,\mu }>24$ GeV) also have an isolation requirement. Efficiency losses at higher $p_{\text {T}}$ values are recovered by higher threshold triggers ($p_{\text {T}} ^e>60$ GeV or $p_{\text {T}} ^\mu >36$ GeV) without any isolation requirement. The integrated luminosity of the analysed data sample is 20.3 fb$^{-1}$.

**Table 1:** Maximum allowed FCNC $t\rightarrow qZ$BRs as predicted by several models [3–9]

Model:	SM	QS	2HDM	FC 2HDM	MSSM	SUSY	RS
BR$(t\rightarrow qZ)$:	$10^{-14}$	$10^{-4}$	$10^{-6}$	$10^{-10}$	$10^{-7}$	$10^{-6}$	$10^{-5}$

## Simulated samples

In the SM, top quarks are produced at the LHC mainly in pairs, with a predicted $t\bar{t}$ cross section in *pp* collisions at a centre-of-mass energy of $\sqrt{s} = 8 \mathrm{TeV}$ of $\sigma _{t\bar{t}}= 253^{+13}_{-15}$ pb for a top-quark mass of $172.5 \mathrm{GeV}$. The cross section has been calculated at next-to-next-to leading-order (NNLO) in QCD including resummation of next-to-next-to leading logarithmic (NNLL) soft gluon terms with Top++ 2.0 [30–35]. The parton distribution function (PDF) and $\alpha _\mathrm{S}$ uncertainties are calculated using the PDF4LHC prescription [36] with the MSTW 2008 68 % CL NNLO [37, 38], CT10 NNLO [39, 40] and NNPDF 2.3 5f FFN [41] PDF sets and are added in quadrature to the renormalisation and factorisation scale uncertainties. The cross-section value for the NNLO+NNLL calculation is about 3 % larger than the exact NNLO prediction implemented in HATHOR 1.5 [42].

The simulation of signal events is performed with PROTOS 2.2 [43, 44], which includes the effects of new physics at an energy scale $\Lambda$ by adding dimension-six effective terms to the SM Lagrangian. The most general *Ztu* vertex that arises from the dimension-six operators can be parameterised including only $\gamma ^\mu$ and $\sigma ^{\mu \nu }q_{\nu }$ terms [45] as:

1 $$\begin{aligned} \mathcal {L}_{Ztu}= & {} -\frac{g}{2c_W}\bar{u}\gamma ^{\mu }\left( X^\mathrm{L}_{ut}P_\mathrm{L}+X^\mathrm{R}_{ut}P_\mathrm{R}\right) tZ_{\mu }\nonumber \\&-\frac{g}{2c_W}\bar{u}\frac{i\sigma ^{\mu \nu }q_{\nu }}{m_Z}\left( \kappa ^\mathrm{L}_{ut}P_\mathrm{L}+\kappa ^\mathrm{R}_{ut}P_\mathrm{R}\right) tZ_{\mu } + \mathrm {h.c.}, \end{aligned}$$

where *g* is the electroweak coupling, $c_W$ is the cosine of the weak mixing angle, *u* and *t* are the quark spinors, $Z_\mu$ is the *Z* boson field, $P_{\mathrm {L}}$ ($P_{\mathrm {R}}$) is the left-handed (right-handed) projection operator, $m_Z$ is the *Z* boson mass and $q^{\nu }=p^{\nu }_t-p^{\nu }_u$ is the outgoing *Z* boson momentum. The *Ztc* vertex can be parameterised in a similar fashion. This vertex involves a minimum of four anomalous couplings $X^{\mathrm {L}}_{ut}, X^{\mathrm {R}}_{ut}, \kappa ^L_{ut},\kappa ^R_{ut}$, which are set to 0.01 each. It was checked that the coupling choice does not affect the kinematics of the event. No impact in the kinematics is seen by comparing the *bWuZ* and *bWcZ* processes and the latter is used as reference. Only decays of the *W* and *Z* bosons involving charged leptons are generated at the matrix-element level by PROTOS ($Z\rightarrow e^+e^-,\mu ^+\mu ^-,\tau ^+\tau ^-$ and $W\rightarrow e\nu ,\mu \nu ,\tau \nu$). The CTEQ6L1 [46] leading-order PDF is used. To account for higher-order contributions in the signal production, the events are reweighted to the measured $t\bar{t}$ differential cross section as a function of the transverse momentum of the $t\bar{t}$ system $(1/\sigma )(\mathrm {d}\sigma /\mathrm {d}p_{\mathrm {T}}^{t\bar{t}})$ [47]. Hadronisation is handled by PYTHIA 6.426 [48] with the Perugia2011C [49] set of tuned parameters and $\tau$ decays are processed with TAUOLA [50]. The top-quark mass is set to $m_t = 172.5$ GeV. Additional simulations with different parton shower parameterisations are used to estimate the systematic uncertainties on the amount of initial- and final-state radiation (ISR/FSR).

Several SM processes have final-state topologies similar to the signal, with at least three prompt charged leptons, especially *WZ*, *ZZ*, $t\bar{t}V$,^2^$t\bar{t}H$, *ggH*, *VH*, *tZ* and triboson (*WWW*, *ZWW* and *ZZZ*) production. Events with non-prompt leptons or in which at least one jet is misidentified as an isolated charged lepton (labelled as “fake leptons” throughout this paper) can also fulfil the event selection requirements. These events, typically $Z+$ jets, $Z+\gamma$, $t\bar{t}$ and single-top, are estimated from a data-driven method using a parameterisation of the true- and fake-lepton efficiencies. Samples of simulated events of these backgrounds with fake leptons are used to cross-check the data-driven estimation. The $Z+$jets simulations include *Z* production in association with heavy-flavour quarks.

Table 2 summarises the information about the generators, parton shower and parton distribution functions used to simulate the different event samples considered in the analysis. Diboson events (*WZ* and *ZZ*, where *Z* means $Z/\gamma ^*$) produced using SHERPA contain up to three additional partons and are selected to have leptons with $p_{\text {T}} > 5$ GeV and $m_{\ell \ell } > 0.1$ GeV for the $Z/\gamma ^*$. The additional *WZ* and *ZZ* samples are used for comparison. The *WZ*ALPGEN samples are simulated with up to five additional partons from the matrix element. The *ZZ*HERWIG [51] samples are selected to have one lepton with $p_{\text {T}} > 10$ GeV and $|\eta | < 2.8$. The simulations of $t\bar{t}Z$, $t\bar{t}W$(*W*), *tZ* and tribosons include events with up to two extra partons in the final state. The simulated samples used to cross-check the data-driven estimation of background with fake leptons are also listed in Table 2.

Detailed and fast simulations of the detector and trigger are performed with standard ATLAS software using GEANT4 [52, 53] and ATLFASTII [53], respectively. The same offline reconstruction methods used on data are applied to the simulated samples.

**Table 2:** Generators, parton shower, parton distribution functions and parameter tune for hadronisation used to produce simulated samples used in this analysis

Sample	Generator	Parton shower	PDF	Tune
Samples with at least three prompt leptons				
$t\bar{t} \rightarrow bWqZ$	PROTOS 2.2 [43]	PYTHIA 6.426 [48]	CTEQ6L1 [46]	Perugia2011C [49]
*WZ*	SHERPA 1.4.3 [54]	SHERPA 1.4.3	CT10 [39]	–
*WZ*	ALPGEN 2.14 [55]	HERWIG 6.520.2 [51]	CTEQ6L1	AUET2 [56]
*ZZ*	SHERPA 1.4.3	SHERPA 1.4.3	CT10	–
*ZZ*	HERWIG 6.5	HERWIG 6.5	CTEQ6L1	AUET2
$t\bar{t} V$, *tZ*, tribosons	MADGRAPH 5 1.3.33 [57]	PYTHIA 6.426	CTEQ6L1	AUET2B
$t\bar{t} H$, *WH*, *ZH*	PYTHIA 8.163 [58]	PYTHIA 8.163	CTEQ6L1	AU2 [59]
*ggH*	POWHEG 2 [60]	PYTHIA 8.163	CT10	AU2
Other samples				
*WW*	SHERPA 1.4.3	SHERPA 1.4.3	CT10	–
$Z+$ jets (30 GeV $< m_{\ell \ell } < 1$ TeV)	ALPGEN 2.14	PYTHIA 6.426	CTEQ6L1	Perugia2011C
$Z+$ jets (10 GeV $< m_{\ell \ell } < 60$ GeV)	ALPGEN 2.14	HERWIG 6.520.2	CTEQ6L1	AUET2
$Z\gamma$	SHERPA 1.4.1	SHERPA 1.4.1	CT10	–
$t\bar{t} \rightarrow bWbW$	POWHEG 2	PYTHIA 6.426	CTEQ6L1	Perugia2011C
Single top (*s*, *Wt* channel)	MC@NLO 4.03 [61]	HERWIG 6.520.2	CT10	AUET2
Single top (*t* channel)	AcerMC 3.8 [62]	PYTHIA 6.426	CTEQ6L1	AUET2B

## Object reconstruction

The primary physics objects considered in this search are electrons, muons, $E_{\text {T}}^{\text {miss}}$, jets, and *b*-tagged jets. Tau leptons are not explicitly reconstructed, although the $\tau$ decay products are reconstructed as electrons, muons or jets and as an additional contribution to the missing transverse momentum.

Electron candidates are reconstructed [63] from energy deposits (clusters) in the electromagnetic calorimeter, which are then matched to reconstructed charged-particle tracks in the inner detector. The candidates are required to have a transverse energy $E_{\text {T}}$ greater than 15 GeV and a pseudorapidity of the calorimeter cluster associated with the electron candidate $|\eta _{\mathrm {cluster}}|<2.47$. Candidates in the transition region between the barrel and endcap calorimeters with $1.37 < |\eta _{\mathrm {cluster}}| < 1.52$ are excluded. Electron candidates in this analysis must satisfy tight quality requirements on the electromagnetic cluster and associated track which provide discrimination between isolated electrons and jets. In order to suppress multi-jet backgrounds, it is also required that there is little activity in the space surrounding the electron. Two isolation variables are employed: the energy deposited around the electron in the calorimeter in a cone of size $\Delta R = 0.2$ and the scalar sum of the $p_{\text {T}}$ of the tracks within cone of size $\Delta R = 0.3$ around the electron. Cuts on these two quantities are used to select isolated electrons; the adopted cuts yield a 90 % identification efficiency in *Z* boson decays to $e^+e^-$ events from the full 2012 dataset. Additionally, the longitudinal impact parameter $|z_0|$ of the electron track with respect to the selected primary vertex of the event is required to be less than 2 mm. The closest jet if separated by $\Delta R < 0.2$ from the selected electron is removed from the event. The electron candidate is discarded if an additional selected jet is found with $\Delta R < 0.4$. A looser electron selection, used for the estimation of backgrounds with fake leptons, is defined by removing the isolation requirements.

The muon candidate reconstruction [64] is performed by finding, combining and fitting track segments in the layers of the muon chambers, starting from the outermost layer. The identified muons are then matched with tracks reconstructed in the inner detector. The candidates are refitted using the complete track information from both detector systems, and are required to satisfy $p_{\text {T}} > 15$ GeV, $|\eta |<2.5$ and to be separated by $\Delta R > 0.4$ from any selected jet. The hit pattern in the inner detector is required to be consistent with a well-reconstructed track and the $|z_0|$ of the muon track is required to be less than 2 mm. Additionally, the sum of the momenta of tracks inside a cone around the muon candidate, with variable size such that it is smaller for higher muon $p_{\text {T}}$  [65], must be less than 5 % of the muon energy. For the estimation of backgrounds with fake leptons, a looser selection is applied by removing the isolation requirement.

Jets are reconstructed [66] from topological clusters of neighbouring calorimeter cells with significant energy deposits using the anti-*k*$_t$ algorithm [67] with a radius parameter $R=0.4$. Prior to jet finding, a local calibration scheme is applied to correct the topological cluster energies for the non-compensating response of the calorimeter, dead material, and energy leakage. The corrections are obtained from simulations of charged and neutral particles. These jets are then calibrated to the hadronic energy scale using $p_{\text {T}}$- and $\eta$-dependent correction factors. Dedicated requirements are applied to remove the negligible fraction of events (less than 0.01 %) where a jet is incorrectly reconstructed from a few noisy calorimeter cells [68]. The jets used in the analysis are required to have $p_{\text {T}} >25$ GeV and $|\eta |<2.5$. To reduce the number of selected jets that originate from secondary *pp* interactions, for jets with $p_{\text {T}} < 50$ GeV and $|\eta |<2.4$, the scalar sum of the $p_{\text {T}}$ of tracks matched to a jet and originating from the primary vertex must be at least 50 % of the scalar sum of the $p_{\text {T}}$ of all tracks matched to the jet.

Jets containing *b*-hadrons are identified (‘*b*-tagged’) [69] using an algorithm based on multivariate techniques. It combines information from the impact parameters of displaced tracks and from topological properties of secondary and tertiary decay vertices reconstructed within the jet. It is determined with simulated $t\bar{t}$ events that, for the chosen working point, the tagging efficiency for *b*-jets with $p_{\text {T}} >20$ GeV is 70 %, while the rejection factors for light-quark or gluon jets (light jets), charm jets and $\tau$ leptons are 137, 5 and 13, respectively.

The measurement of $E_{\text {T}}^{\text {miss}}$ is based [70] on the energy deposits in the calorimeter with $|\eta |<4.9$. The energy deposits associated with reconstructed jets and electrons are calibrated accordingly. Energy deposits not associated with a reconstructed object are calibrated according to their energy sharing between the electromagnetic and hadronic calorimeters. The momentum associated with each reconstructed muon, estimated using the momentum measurement of its reconstructed track, is taken into account in the calculation of $E_{\text {T}}^{\text {miss}}$.

## Event selection and kinematics

At least one of the selected leptons must be matched, with $\Delta R < 0.15$ to the appropriate trigger object and have $p_{\text {T}} > 25$ GeV. The trigger efficiencies for the leptons are approximately 93 % for electrons, 70 % for muons with $|\eta | < 1.05$ and 86 % for muons with $1.05 < |\eta | < 2.4$ [71, 72]. The events are required to have at least one primary vertex with more than four associated tracks, each with $p_{\text {T}} >400$ MeV. The primary vertex is chosen as the one with the highest $\sum p_{\text {T}} ^2$ over all associated tracks. Leptons from cosmic rays are rejected by removing muon pairs with large, oppositely signed transverse impact parameters ($|d_0|>0.5$ mm) and consistent with being back-to-back in the $r-\phi$ plane. Events with noise bursts and readout errors in the LAr calorimeter are also rejected. Exactly three isolated leptons associated with the same vertex are required. The three leptons must have $|\eta |<2.5$ and $p_{\text {T}} > 15$ GeV. Two of the leptons are required to have the same flavour, opposite charge and a reconstructed mass within 15 GeV of the *Z* boson mass ($m_Z$) [1]. If more than one compatible lepton-pair is found, the one with the reconstructed mass closest to $m_Z$ is chosen as the *Z* boson candidate. According to the signal topology, the events are then required to have $E_{\text {T}}^{\text {miss}} >20$ GeV and two jets, although an additional third jet from initial- or final-state radiation is allowed. All jets are required to have $p_{\text {T}} > 35$ GeV and $|\eta |<2.5$. One or two of the jets must be *b*-tagged. Only one *b*-tagged jet is expected in the signal events, nevertheless a second one can arise from a misidentified *c*-jet associated with the FCNC decay of the top quark. Allowing for the additional *b*-tagged jet increases the signal efficiency without compromising the signal-to-background ratio.

Applying energy–momentum conservation, the kinematics of the top quarks can be reconstructed from the corresponding decay particles. Since the neutrino from the semileptonic decay of the top quark ($t\rightarrow bW\rightarrow b\ell \nu$) is undetected, its four-momentum must be estimated. This can be done by assuming that the lepton not previously assigned to the *Z* boson and the *b*-tagged jet (labelled *b*-jet) originate from the *W* boson and SM top-quark decays, respectively, and that $E_{\text {T}}^{\text {miss}}$ is the neutrino’s transverse momentum. The longitudinal component of the neutrino’s momentum ($p^{\nu }_z$) is then determined by minimising, without constraints, the following expression:

2 $$\begin{aligned} \chi ^2 = \frac{\left( m^{\mathrm {reco}}_{j_a\ell _a\ell _b}-m_{t_{\mathrm {FCNC}}}\right) ^2}{\sigma _{t_{\mathrm {FCNC}}}^2}+ \frac{\left( m^{\mathrm {reco}}_{j_b\ell _c\nu }-m_{t_{\mathrm {SM}}}\right) ^2}{\sigma _{t_{\mathrm {SM}}}^2} + \frac{\left( m^{\mathrm {reco}}_{\ell _c\nu }-m_W\right) ^2}{\sigma _W^2}, \end{aligned}$$

where $m^{\mathrm {reco}}_{j_a\ell _a\ell _b}$, $m^{\mathrm {reco}}_{j_b\ell _c\nu }$ and $m^{\mathrm {reco}}_{\ell _c\nu }$ are the reconstructed masses of the *qZ*, *bW* and $\ell \nu$ systems, respectively. The central value for the masses and the widths of the top quarks and *W* boson are taken from reconstructed simulated signal events. This is done by matching the true particles in the simulated events to the reconstructed ones, setting the longitudinal momentum of the neutrino to the $p_z$ of the true simulated neutrino and then performing Bukin fits^3^ [73] to the masses of the matched reconstructed top quarks and *W* boson. The values are $m_{t_{\mathrm {FCNC}}}=173$ GeV, $\sigma _{t_{\mathrm {FCNC}}}=10$ GeV, $m_{t_{\mathrm {SM}}}=168$ GeV, $\sigma _{t_{\mathrm {SM}}}=23$ GeV, $m_W=82$ GeV and $\sigma _W=15$ GeV.

**Fig. 1:**
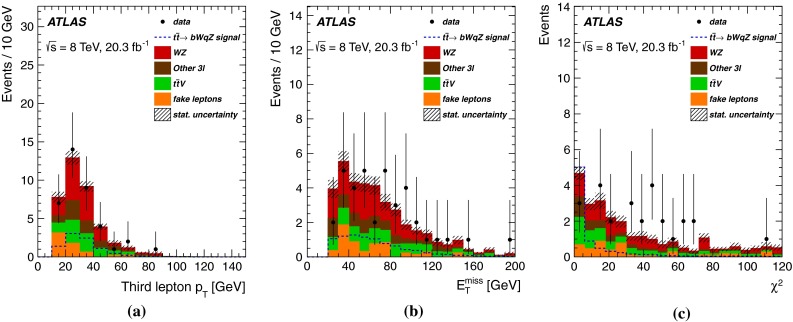
Expected (*filled histogram*) and observed (*points* with *error bars*) distributions in the signal region before the $\chi ^2$ cut is applied for (**a**) $p_{\text {T}}$ of the third lepton, **b** $E_{\text {T}}^{\text {miss}}$ and (**c**) $\chi ^2$ of the kinematics reconstruction. For comparison, distributions for the FCNC $t\bar{t} \rightarrow bWqZ$ signal (*dashed line*), normalised to the observed 95 % CL limit reported in this paper, are also shown. Background statistical uncertainties associated with the number of events in the samples are represented by the hatched areas

For each jet combination, where any jet can be assigned to $j_a$, while $j_b$ must correspond to a *b*-tagged jet, the $\chi ^2$ minimisation gives the most probable value for $p^{\nu }_z$. From all combinations, the one with the minimum $\chi ^2$ is chosen, along with the corresponding $p^{\nu }_z$ value. The jet from the top-quark FCNC decay is referred to as the light-quark (*q*) jet. The fractions of correct assignments between the reconstructed top quarks and the true simulated particles (evaluated as a match within a cone of size $\Delta R = 0.4$) are $\epsilon _{t_{\mathrm {FCNC}}}=79.9$ % and $\epsilon _{t_{\mathrm {SM}}}=56.3$ %. Figure 1 shows the $p_{\text {T}}$ of the third lepton as well as the $E_{\text {T}}^{\text {miss}}$ and $\chi ^2$ distributions at this level of the analysis.

The selection of the signal region is concluded with the requirement of $\chi ^2<6$, which optimises the sensitivity discussed in Sect. 8.

## Background estimates

Three control regions are defined to check the agreement between data and simulated samples of the *ZZ*, *WZ* and $t\bar{t}Z$ backgrounds. No scaling factors are derived from these control regions, however they are used to estimate the background modelling uncertainties described in Sect. 7. The *tZ* contribution to the total background is expected to be smaller than the one from $t\bar{t}Z$ events [74]. Due to the similarity between the final states of *tZ* and signal events, there are large signal contributions to possible *tZ* control regions. For these reasons no control region is defined for the *tZ* background.

The *ZZ* control region is defined by requiring two pairs of leptons with the same flavour, opposite charge and a reconstructed mass within 15 GeV of the *Z* boson mass. The expected and observed yields are shown in Table 3 and the SHERPA sample is chosen as reference.

**Table 3:** Event yields in the *ZZ* control region for all significant sources of background. The *ZZ* SHERPA sample is taken as reference for the total background estimation. The first uncertainty is the statistical one associated with the number of events in the simulated samples, the second uncertainty is systematic and is described in Sect. 7. The entry labelled “other backgrounds” includes all the remaining backgrounds described in Sect. 3 and in Table 2. The signal efficiency is also shown

Sample	Yields
*ZZ* (SHERPA)	$87\pm 4\pm 5$
*ZZ* (HERWIG)	$85\pm 4\pm 5$
Other backgrounds	$0.48\pm 0.05\pm 0.08$
Total background	$88\pm 4\pm 5$
Data	95
Signal efficiency $$(\times $$ $$10^{-8})$$	$$5.6\pm 4.3\pm 0.1$$

To study the *WZ* background the following control region is defined. Events are required to have three leptons, two of them with the same flavour, opposite charge and a reconstructed mass within 15 GeV of the *Z* boson mass. Additional requirements include the presence of at least one jet in the event with $p_{\text {T}} > 35$ GeV, no *b*-tagged jets with $p_{\text {T}} > 35$ GeV and a *W* boson transverse mass, built with the residual lepton and $E_{\text {T}}^{\text {miss}}$, greater than 50 GeV. Table 4 shows the expected and observed yields in this control region. The best estimation comes from the SHERPA prediction, which is chosen as the reference sample.

**Table 4:** Event yields in the *WZ* control region for all significant sources of background. The *WZ* SHERPA sample is taken as reference for the total background estimation. The first uncertainty is the statistical one associated with the number of events in the samples, the second uncertainty is systematic and is described in Sect. 7. The entry labelled “other backgrounds” includes all the remaining backgrounds described in Sect. 3 and in Table 2. The signal efficiency is also shown

Sample	Yields
*WZ* (SHERPA)	$333\pm 5\pm 17$
*WZ* (ALPGEN)	$393\pm 6\pm 19$
*ZZ*	$35\pm 3\pm 6$
Fake leptons	$15\pm 3\pm 5$
Other backgrounds	$9.5\pm 0.3\pm 2.4$
Total background	$392\pm 7\pm 19$
Data	405
Signal efficiency $(\times 10^{-4})$	$9.8\pm 0.1\pm 1.0$

The $t\bar{t}Z$ control region is defined by requiring at least three leptons, two of them with the same flavour, opposite charge and a reconstructed mass within 15 GeV of the *Z* boson mass. Furthermore the events are required to have at least two jets with $p_{\text {T}} > 25$ GeV and at least two *b*-tagged jets if there are three leptons in the event, or at least one *b*-tagged jet if there are four or more leptons in the event. Since the signal contribution for events with three leptons and two *b*-tagged jets is small, the overlap between signal and background regions is not removed, increasing the $t\bar{t}Z$ sensitivity in this control region. Table 5 shows the yields in this control region, and the background yields agree very well with the data within the given uncertainty.

**Table 5:** Event yields in the $t\bar{t}Z$ control region for all significant sources of background. The first uncertainty is the statistical one associated with the number of events in the samples, the second uncertainty is systematic and is described in Sect. 7. The entry labelled “other backgrounds” includes all the remaining backgrounds described in Sect. 3 and in Table 2. The signal efficiency is also shown

Sample	Yields
$t\bar{t}V$	$8.3\pm 0.2\pm 2.7$
*tZ*	$2.0\pm 0.1\pm 1.0$
*WZ*	$1.8\pm 0.3\pm 0.4$
Other backgrounds	$1.8\pm 0.4\pm 0.4$
Total background	$13.9\pm 0.6\pm 3.0$
Data	12
Signal efficiency $(\times 10^{-4})$	$3.9\pm 0.1\pm 0.6$

Backgrounds from events which contain at least one fake lepton are estimated from data using the matrix method [75]. This is based on the measurement of the efficiencies of real and fake loose leptons to pass the nominal selection, $\epsilon _\mathrm{R}$ and $\epsilon _\mathrm{F}$, and on the selection of two orthogonal sets of events in the signal region. For the first of these sets, the nominal requirements are used for the leptonic selection, while for the second one, only the leptons which satisfy the looser selection (as described in Sect. 4) but without meeting the nominal requirements are considered. For the single-lepton case, the number of events with one fake nominal lepton is $N^{\mathrm {nominal}}_\mathrm{F} = \left( \epsilon _\mathrm{F}/\epsilon _\mathrm{R} - \epsilon _\mathrm{F}\right) \left( (\epsilon _\mathrm{R}-1)N_\mathrm{T} + \epsilon _\mathrm{R}N_\mathrm{L}\right)$, where $N_\mathrm{T}$ ($N_\mathrm{L}$) represents the number of selected events in the first (second) set defined above. The method is extrapolated to the three-lepton topology, with a $8\times 8$ matrix that is inverted using a numerical method to obtain the number of events with at least one fake lepton. The efficiencies for real and fake leptons are estimated as a function of the lepton transverse momentum by a fit of the matrix method results to two dedicated enriched samples of real and fake leptons: a sample of $Z\rightarrow \ell ^+\ell ^-,\ell =e,\mu$ and a same-sign dilepton sample (excluding same-flavour events with a reconstructed mass compatible with a *Z* boson). In both samples, in order to improve the modelling of fake leptons originating from heavy-flavour decays, only events with at least one additional *b*-tagged jet are considered. The efficiency $\epsilon _\mathrm{R}$ ranges from 0.74 to 0.88 (0.80–0.99) and $\epsilon _\mathrm{F}$ from 0.010 to 0.13 (0.035–0.18) for electrons (muons). The relevant uncertainties are calculated from the discrepancy between predicted and observed number of events in the control region detailed below.

A control region to test the performance of the fake-lepton estimation method and derive its uncertainty is defined. It requires three leptons with $p_{\text {T}} < 50$ GeV (the third one with $p_{\text {T}} <30$ GeV), two of them having the same flavour, opposite charge and a reconstructed mass within 15 GeV of the *Z* boson mass, at least one *b*-tagged jet with $p_{\text {T}} > 35$ GeV and $E_{\text {T}}^{\text {miss}} < 40$ GeV. As for the $t\bar{t}Z$ control region, there is a small overlap with the signal region, which is not removed in order to increase the sensitivity to the fake-lepton backgrounds. The yields are shown in Table 6 and agree well between data and expectation. As a validation of the matrix method, the background in which exactly one of the leptons is a fake lepton is also evaluated using simulated samples. The results for the signal region and different control regions are consistent between the two methods within the estimated uncertainties.

**Table 6:** Event yields in the fake-lepton control region for all significant sources of background. The first uncertainty is the statistical one associated with the number of events in the samples, the second uncertainty is systematic and is described in Sect. 7. The entry labelled “other backgrounds” includes all the remaining backgrounds described in Sect. 3 and in Table 2. The signal efficiency is also shown

Sample	Yields
Fake leptons	$7\pm 1\pm 4$
*WZ*	$2.7\pm 0.4\pm 0.7$
*ZZ*	$1.7\pm 0.6\pm 0.8$
Other backgrounds	$1.7\pm 0.1\pm 0.6$
Total background	$13\pm 1\pm 4$
Data	17
Signal efficiency $[\times 10^{-4}]$	$1.77\pm 0.06\pm 0.20$

**Fig. 2:**
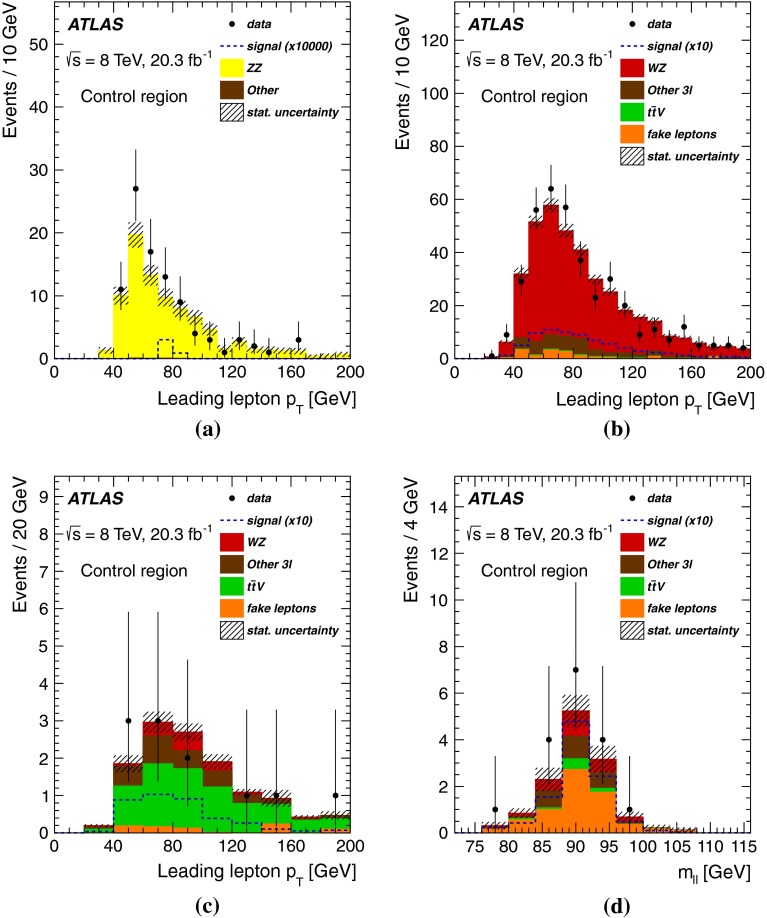
Expected (*filled histogram*) and observed (*points* with*error bars*) distributions for the $p_{\text {T}}$ of the leading lepton in the **a** *ZZ*, **b** *WZ* and **c** $t\bar{t}Z$ control regions and **d** reconstructed mass of the two leptons with the same flavour and opposite charge in the fake-lepton control region. For comparison, distributions for the FCNC $t\bar{t} \rightarrow bWqZ$ signal (*dashed line*), scaled to $10^4$ or 10 times the observed 95 % CL limit, are also shown. Background statistical uncertainties associated with the number of events in the samples are represented by the *hatched areas*

Figure 2 shows the $p_{\text {T}}$ of the leading lepton for the *ZZ*, *WZ* and $t\bar{t}Z$ control regions, and the reconstructed mass of the two leptons with the same flavour and opposite charge for the fake-lepton control region.

## Systematic uncertainties

The effect of each source of systematic uncertainty is studied by independently varying the corresponding central value and propagating this through the full analysis chain. The relative impact of each type of systematic uncertainty on the total background and signal is summarised in Table 7.

The main uncertainty on the backgrounds comes from their modelling, which has the following two contributions. The level of agreement with data of the reference samples is assessed from the combination of the Poisson uncertainty on the available amount of data with the statistical uncertainty of the expected background yields in the dedicated control regions described in Sect. 6. The uncertainties are estimated to be 6.3, 12, 42 and 62 %, for the *WZ*, *ZZ*, $t\bar{t}Z$ and fake-lepton backgrounds, respectively. The other contribution comes from the uncertainty on the theoretical prediction in the signal region and is estimated using the alternative *WZ* and *ZZ* simulated samples. The corresponding uncertainties are 17 and 100 %, respectively. Similarly, for $t\bar{t}Z$, *tZ* and Higgs samples, conservative values of 30 % [76, 77], 50 % [74] and 15 % [78] respectively, are used, in order to account for the theoretical uncertainties. The combination of all these uncertainties gives a 17% effect on the total background estimation.

The theoretical uncertainties of the signal modelling, as described in Sect. 3, namely production cross section and ISR/FSR modelling, are found to be 5.5 %.

For both the estimated signal and background event yields, experimental uncertainties resulting from detector effects are considered. The lepton reconstruction, identification and trigger efficiencies, as well as lepton momentum scales and resolutions [63, 79, 80] are considered. The overall effect on the total background yield and the signal efficiency is estimated to be 4.7 and 2.9 % respectively. The effect of the jet energy scale and resolution [66, 81] uncertainties are evaluated as 7.7 and 4.9 % for the background and signal, respectively. The *b*-tagging performance component, which includes the uncertainty of the *b*-, *c*-, mistagged- and $\tau$-jet scale factors (the $\tau$ and charm uncertainties are highly correlated and evaluated as such) is evaluated by varying the $\eta$-, $p_{\text {T}}$- and flavour-dependent scale factors applied to each jet in the simulated samples. It is estimated to be 3.9 % for the total background and 7.2 % for the signal efficiency. The $E_{\text {T}}^{\text {miss}}$ scale uncertainty [70] is found to vary the total background yield and the signal efficiency by 3.2 and 1.5 %, respectively. All these detector systematic uncertainties are treated as fully correlated between signal and background.

The uncertainty related to the integrated luminosity for the dataset used in this analysis is 2.8 %. It is derived following the methodology described in Ref. [82]. It only affects the estimations obtained from simulated samples, therefore its impact on the total background yield estimation is 2.4 %.

**Table 7:** Summary of the impact of each type of uncertainty on the total background and signal yields. The values are shown as the relative variations from the nominal values. The statistical uncertainty associated with the number of events in the simulated samples is also shown

Source	Background (%)	Signal (%)
Background modelling	17	–
Signal modelling	–	5.5
Leptons	4.7	2.9
Jets	7.7	4.9
*b*-Tagging	3.9	7.2
$E_{\text {T}}^{\text {miss}}$	3.2	1.5
Luminosity	2.4	2.8
Statistical	8.1	1.5

## Results

**Fig. 3:**
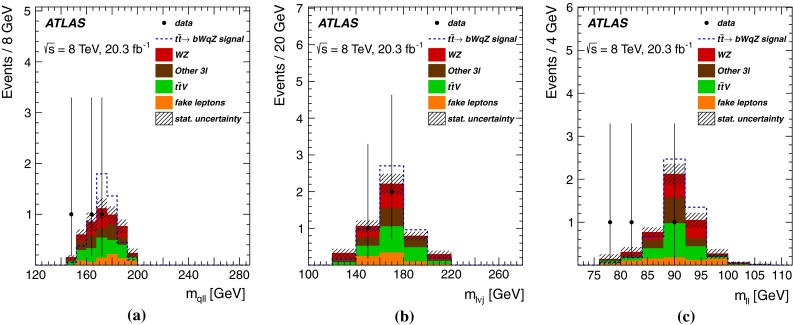
Expected (*filled histogram*) and observed (*points* with *error bars*) distributions in the signal region after the final selection is applied for the reconstructed masses of the **a** top quark from the FCNC decay, **b** top quark from the SM decay and **c** *Z* boson. For comparison, distributions for the FCNC $t\bar{t} \rightarrow bWqZ$ signal (*dashed line*), normalised to the observed 95 % CL limit, are also shown. Background statistical uncertainties are represented by the *hatched areas*

Table 8 shows the expected number of background events, number of selected data events and signal efficiency after the final event selection described in Sect. 5. Figure 3 shows the reconstructed masses of the top quarks and *Z* boson after the final selection. Good agreement between data and background yields is observed at all stages of the analysis. No evidence for the $t\rightarrow qZ$ decay is found and a 95 % CL upper limit on the number of signal events is derived using the modified frequentist likelihood method [83, 84].

**Table 8:** Expected number of background events, number of selected data events and signal efficiency (normalised to all decays of the *W* and *Z* bosons), after the final selection. The first uncertainty is the statistical one associated with the number of events in the samples, the second uncertainty is systematic and is described in Sect. 7. The entry labelled “other backgrounds” includes all the remaining backgrounds described in Sect. 3 and in Table 2

Sample	Yields
*WZ*	$1.3\pm 0.2\pm 0.6$
$t\bar{t}V$	$1.5\pm 0.1\pm 0.5$
*tZ*	$1.0\pm 0.1\pm 0.5$
Fake leptons	$0.7\pm 0.3\pm 0.4$
Other backgrounds	$0.2\pm 0.1\pm 0.1$
Total background	$4.7\pm 0.4\pm 1.0$
Data	3
Signal efficiency $(\times 10^{-4})$	$7.8\pm 0.1\pm 0.8$

The test-statistic $X_d$, which compares the number of observed data events with background and signal expectations, is defined as:

3 $$\begin{aligned} X_d=n\ln \left( 1+\frac{s}{b}\right) \end{aligned}$$

where *n*, *s* and *b* are the numbers of data, expected background and signal events, respectively. The $X_d$ statistical test is then compared to $10^5$ pseudo-experiments for the hypotheses of signal plus background ($X_{s+b}$) and background-only ($X_b$), which are obtained by replacing *n* with the corresponding number of events produced by each pseudo-experiment. The statistical fluctuations of the pseudo-experiments are implemented assuming that the number of events follows a Poisson distribution. All statistical and systematic uncertainties on the expected backgrounds and signal efficiencies, as described in Sect. 7, are taken into account and implemented assuming Gaussian distributions.

The CL for a given signal hypothesis *s* is defined as [83]:

4 $$\begin{aligned} 1-\mathrm {CL}={\int _0^{X_d}P_{s+b}(X)\mathrm {d}X \over \int _0^{X_d}P_{b}(X)\mathrm {d}X}\, , \end{aligned}$$

where $P_{s+b}$ and $P_b$ are the probability density functions obtained from the pseudo-experiments for the $X_{s+b}$ and $X_b$ values, respectively, and are functions of *s* and *b*. The limit on the number of signal events is determined by finding the value of *s* corresponding to a CL of 95 %. The expected limit is computed by replacing $X_d$ with the median of the statistical test for the background hypothesis ($X_b$).

The limits on the number of signal events are converted into upper limits on the $t\rightarrow qZ$ branching fraction using the NNLO + NNLL calculation, and uncertainty, for the $t\bar{t}$ cross section, and constraining BR$(t\rightarrow bW)=1-\mathrm {BR}(t\rightarrow qZ)$. Table 9 shows the observed limit on BR$(t\rightarrow qZ)$ together with the expected limit and corresponding $\pm 1\sigma$ bounds. These values are calculated using the reference $t\bar{t}\rightarrow bWcZ$ sample, since it gives a more conservative result than the $t\bar{t}\rightarrow bWuZ$ sample. The smaller *b*-tagged jet multiplicity of the $t\bar{t}\rightarrow bWuZ$ signal sample leads to an improvement of 4 % in the limit.

**Table 9:** 95 % CL limits Observed and expected 95 % CL limits on the FCNC top-quark decay BRs. The expected central value is shown together with the $\pm 1\sigma$ bands, which includes the contribution from the statistical and systematic uncertainties

Observed	$7\times 10^{-4}$
$(-1\sigma )$	$6\times 10^{-4}$
Expected	$8\times 10^{-4}$
$(+1\sigma )$	$12\times 10^{-4}$

**Fig. 4:**
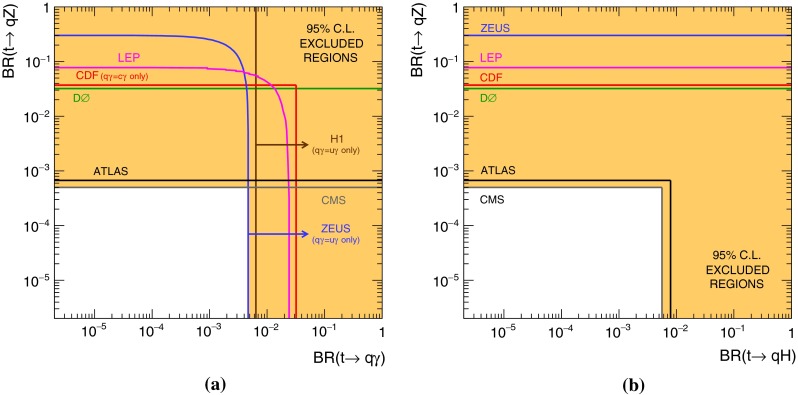
The current 95 % CL observed limits on the **a** BR$(t\rightarrow q\gamma )$ vs BR$(t\rightarrow qZ)$ and **b** BR$(t\rightarrow qH)$ vs BR$(t\rightarrow qZ)$ planes are shown. The *full lines* represent the results from the ATLAS [25], CDF [16, 21] CMS [19, 27], DØ [17], H1 [20], LEP (combined results of the ALEPH, DELPHI, L3 and OPAL collaborations) [10–14] and ZEUS [15] collaborations. The *ATLAS lines* correspond to the limit on BR($t\rightarrow qZ$) set in this paper

Figure 4 compares the 95 % CL observed limit found in this analysis with the results from other FCNC searches performed by the H1, ZEUS, LEP (combined results of the ALEPH, DELPHI, L3 and OPAL collaborations), CDF, DØ and CMS collaborations. The results presented in this paper are consistent with the ones from the CMS Collaboration.

## Conclusions

A search for the FCNC top-quark decay $t\rightarrow qZ$ in events with three leptons has been performed using LHC data collected by the ATLAS experiment at a centre-of-mass energy of $\sqrt{s} = 8$ TeV and corresponding to an integrated luminosity of 20.3 fb$^{-1}$ recorded in 2012. No evidence for signal events is found and a 95 % CL limit for the $t\rightarrow qZ$ branching fraction is established at BR$(t\rightarrow qZ) < 7\times 10^{-4}$, in agreement with the expected limit of BR$(t\rightarrow qZ) < 8\times 10^{-4}$.

## Footnotes

1 ATLAS uses a right-handed coordinate system with its origin at the nominal interaction point in the centre of the detector and the *z*-axis along the beam pipe. The *x*-axis points from the interaction point to the centre of the LHC ring, and the *y*-axis points upward. Cylindrical coordinates $(r,\phi )$ are used in the transverse plane, $\phi$ being the azimuthal angle around the beam pipe. The pseudorapidity is defined in terms of the polar angle $\theta$ as $\eta =-\ln \tan (\theta /2)$. The $\Delta R$ distance is defined as $\Delta R=\sqrt{(\Delta \eta )^2+(\Delta \phi )^2}$.

2 *V* stands for *W* and *Z* bosons. The simulation of $t\bar{t}W$ events also includes a possible second *W* boson.

3 This is a piecewise function with a Gaussian function in the centre and two asymmetric tails.
